# Effect of diet protein restriction on progression of chronic kidney disease: A systematic review and meta-analysis

**DOI:** 10.1371/journal.pone.0206134

**Published:** 2018-11-07

**Authors:** Bingjuan Yan, Xiaole Su, Boyang Xu, Xi Qiao, Lihua Wang

**Affiliations:** Renal Division, Shanxi Medical University Second Hospital, Taiyuan, Shanxi, China; University of Mississippi Medical Center, UNITED STATES

## Abstract

**Background:**

Dietary protein restriction has long been thought to play an important role in the progression of chronic kidney disease (CKD); however, the effect of dietary protein on the rate of decline in kidney function remains controversial.

**Objective:**

We undertook a systematic review and meta-analysis of randomized controlled trials (RCTs) to assess the influence of protein restriction on chronic kidney disease.

**Method:**

Ovid MEDLINE (from 1946 to March 5, 2016), EMBASE (from 1966 to March 5, 2016), and the Cochrane Library (Inception to March 5, 2016) were searched to identify RCTs comparing different levels of protein intake for at least 24 weeks in adult patients with CKD. The outcomes included kidney failure events, the rate of change in estimated glomerular filtration rate (eGFR) per year, all cause death events, and changes in proteinuria, serum phosphorus concentration, serum albumin, and body mass index (BMI).

**Results:**

Nineteen trials with 2492 subjects were analyzed. A low protein diet reduced the risk of kidney failure (odds ratio (OR) = 0.59, 95% CI: 0.41 to 0.85) and end-stage renal disease (ESRD) (OR = 0.64, 95% CI: 0.43 to 0.96), but did not produce a clear beneficial effect for all cause death events (OR = 1.17, 95% CI: 0.67 to 2.06). The change in the mean difference (MD) for the rate of decline in the eGFR was significant (MD: −1.85, P = 0.001), and for proteinuria (MD: −0.44, P = 0.02). A low protein diet also reduced the serum phosphorus concentration (MD: −0.37, 95% CI: −0.5 to −0.24) and BMI (MD: −0.61, 95% CI: −1.05 to −0.17). However the change in albumin presented no significant difference between two groups (MD: 0.23, 95% CI: −0.51 to 0.97).

**Conclusions:**

Based on the findings of our meta-analysis, protein-restricted diet may reduce the rate of decline in renal function and the risk of kidney failure for CKD populations, but did not produce a clear beneficial effect for all cause death events. Besides However, the optimal level of protein intake in different participants is left unanswered, and the nutritional status should be regarded with caution.

## Introduction

The high prevalence of chronic kidney disease (CKD) raises concerns worldwide,[1–3] and evidence-based strategies to delay progression have been proposed, while the application of protein restricted diet remains controversial.[4,5] Several meta-analyses of randomized, prospective trials for patients with CKD indicated that low protein diets (LPDs) and supplemented very low protein diets (SVLPDs) delay the composite outcome of death or the onset of renal replacement therapy (RRT).[6,7] Unfortunately, the results of other studies did not consistently show that protein restriction is beneficial in patients with CKD. The original result of ‘Modification of Diet in Renal Disease’ (MDRD) study, which has thus far been the largest controlled trial of dietary protein management in CKD, failed to show the definite effectiveness of LPD in retarding CKD progression (measured with rate of decline in GFR). [8] Especially in patients with diabetic nephropathy and with CKD at the late stage (stage 4, 5, 5D), the benefits of LPD were not confirmed because of conflicting published reports. [9–11]

Furthermore, there is an unfounded concern that LPD and SVLPDs may cause protein-energy malnutrition. Clinical studies have shown that under careful monitoring, skeletal mass, skeletal function, and body composition are preserved in the majority of CKD patients on LPD.[12] In a recent 18-month randomized controlled trial (RCT) comparing ketoanalog-supplemented VLPD with LPD, participants in both groups showed average energy intakes of 30 kcal/kg IBW/day and preserved their nutritional status.[13] In the follow-up study of MDRD, although there were no differences in nutritional parameters between the VLPD *vs*. LPD group.[14] However, inadequate follow-up time, and the lack of measurement of dietary protein intake during the long-term follow-up period may mislead the conclusion.

Presentation of different results mentioned above has led to uncertainty about the presence and magnitude of the kidney protective effects of dietary protein restriction. In addition, whether the benefit of protein restriction is warranted in view of the potential for causing malnutrition remains inconclusive and controversial. The published meta-analysis with RCTs on the topic cannot integrally answer these key questions in clinical practice, such as the kidney failure events, all-caused death, and nutritional status. Incomplete piece of work impels us to conduct the study. With this systematic review, our aim was to synthesize all available RCTs data and evaluate the most likely beneficial uses and potential limitations of dietary protein restriction thoroughly on patients with CKD, especially the hard endpoints, including kidney failure events and all-caused death.

## Methods

### Data sources

This systematic review was performed according to a pre-specified protocol (**S1 Item)** registered at PROSPERO (CRD 42016038121) and was reported in line with the PRISMA guidelines (**S1 Checklist**).[15] Using relevant keywords and medical subject headings that included all spellings of known RCTs, CKD, keto acids and dietary protein restriction, we identified studies by searching the following databases: MEDLINE by the Ovid portal (from 1946 to March 5, 2016), Embase (from 1966 to March 5, 2016), and the Cochrane Library database (Inception to March 5, 2016), (see **S1 Item** for full search terms). Trials were considered without language restriction. The ClinicalTrials.gov website for RCTs and reference lists from the identified trials and review articles were scanned manually to identify any other relevant studies.

### Study selection and outcome measures

We included data from RCTs that compared different levels of protein intake for adult patients with CKD, including those in dialysis. The difference in protein intake between protein restriction and control groups must have been at least 0.2 g/kg/day. To assess the long-term influence of protein restriction on CKD, trials were excluded if their duration was less than 24 weeks.

Predefined outcomes that contained analyzable data were listed as follows. First, kidney failure events, including more than 25% decrease in, or halving of, the estimated glomerular filtration rate (eGFR),[16] doubling of serum creatinine, or end-stage renal disease (ESRD), as defined by the authors of each study during the follow-up period. We pooled eGFR data calculated by the Modification of Diet in Renal Disease study formula, and creatinine clearance (mL/min/1.73 m^2^). Second, the annual rate of change in eGFR. Positive differences represented a slower decline in the treatment group than in the control group. Third, all-cause death. Fourth, change of urinary protein excretion from baseline to the end of follow-up. All available data of proteinuria were from 24-hour urinary protein and a simple unit conversion to grams per 24 hours was done. Fifth, changes in BMI, serum albumin, and phosphorus concentration from baseline to the end of follow-up.

### Data extraction and quality assessment

Published reports were obtained for each eligible trial, and the relevant information was extracted into a spreadsheet. Study characteristics; baseline patient characteristics; expected and actual diet protein intake; follow-up duration; changes in serum creatinine, eGFR, proteinuria, serum albumin concentration and BMI; and outcome events were recorded.

We used the Cochrane Collaboration risk-of-bias tool to assess sources of bias (**S2 Item**),[17] and the jadad scale to quantify the study quality.[18]

Two investigators (X.S. and B.Y.) performed the literature search, study selection, data extraction, and quality assessment independently, according to a pre-defined protocol. We used a kappa statistic to measure agreement between the two reviews for full text inclusion, and the value was 75%, which reflect an excellent agreement. Disagreement was resolved by consensus or by discussion with the third investigator (W.L.).

### Statistical analysis

Individual odds ratios (ORs) and 95% confidence intervals (CIs) for binary outcomes before pooling were calculated if the ORs were unavailable in the original article. Estimates of ORs were obtained with the Der Simonian-Laird random-effects model, [19] in consideration of the potential heterogeneity among the included studies. Mean differences were used to pool all continuous variables, including eGFR, proteinuria, serum albumin, and BMI. Summary estimates of mean differences were also obtained using a random effects model. We used the original data when the data of change were available in the paper. When data for change from baseline were available in the included trials, we directly extracted them from the literature. When the change-from-baseline standard deviation was missing, we calculated it using correlations that were estimated from other included studies that had a similar follow-up period and reported in considerable detail according to the imputed formulation and its related interpretations in Cochrane Handbook.[20]

The following pre-specified sensitivity analyses were carried (**S1 Item**): Using different random-effects estimation methods, including empirical Bayes [21] and restricted maximum likelihood, [22] estimators with the CIs were constructed using the Knapp–Hartung approach; [23] exclusion of trials with smaller sample sizes; exclusion of trials with shorter follow-up duration; exclusion of trials with lower Jadad scores.

The percentage of variability across studies attributable to heterogeneity beyond chance was estimated using the *I*^*2*^ and *tau*^*2*^ statistics. The *I*^*2*^ value of 25%, 50%, 75% respectively represent low, moderate, and high heterogeneity.[17] Pre-specified subgroup analysis was performed to investigate the source of heterogeneity by several major covariates, including baseline mean eGFR, baseline mean proteinuria, mean age, follow-up time, and whether the population was diabetic (**S1 Item**). *Post-hoc* subgroup analysis was conducted based on the level of protein intake in the protein restriction group, and whether the patients have started renal replacement therapy. Chi-squared test and meta-regression were used to assess the between-subgroup heterogeneity.

A two-side *P*-value less than 0.05 was regarded as statistically significant for all analysis. We used STATA software (version 12.0) to perform the statistical analysis. Review Manager (version 5.3) was used to summarize the individual and aggregate risk of bias (**S1 and S2 Figs**).

## Results

### Search results and characteristics of included studies

**Fig 1:**
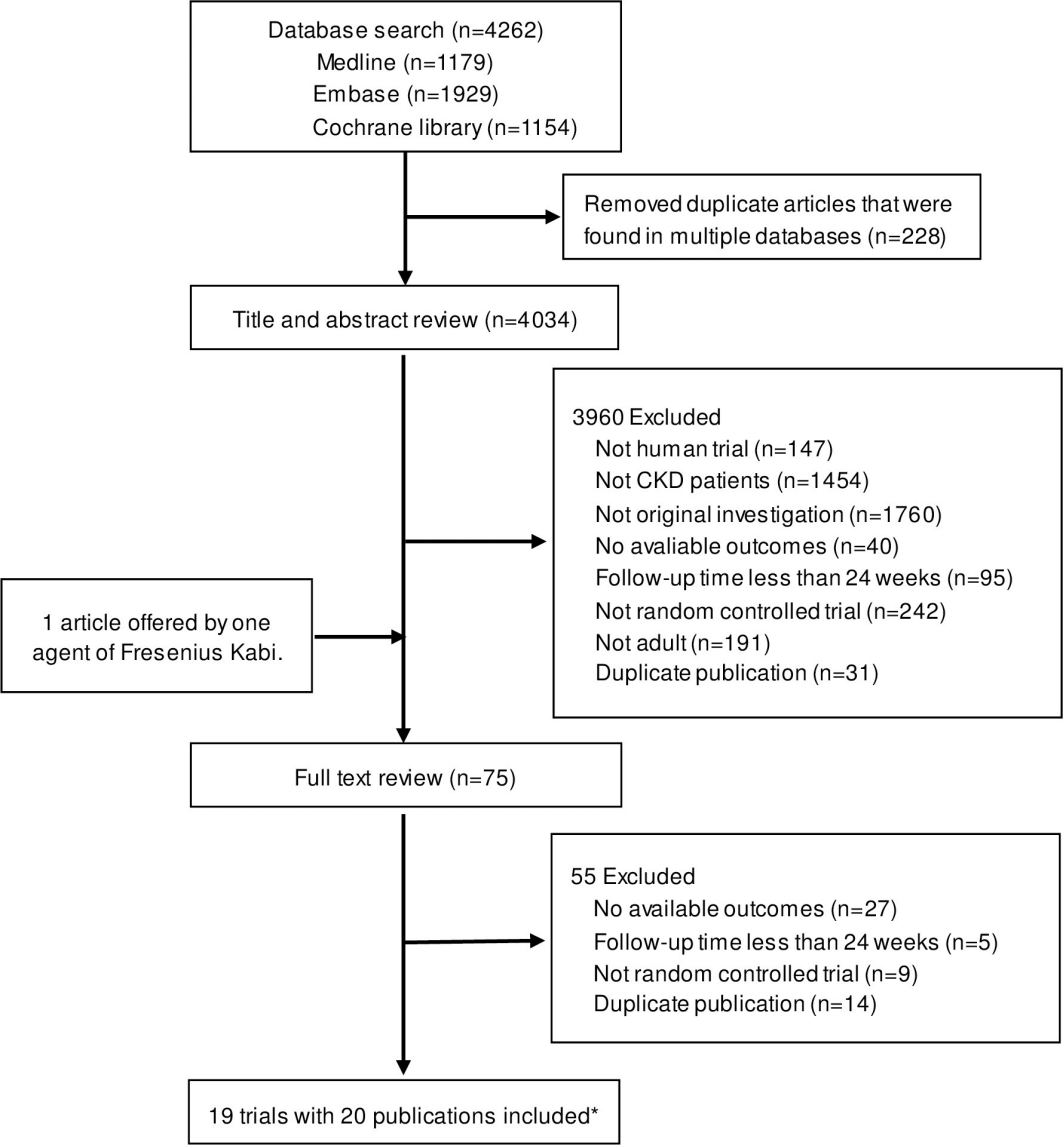
Identification process for eligible studies. *One publication was a post-hoc analysis of MDRD study.

We identified 4262 potentially relevant references from database searches (4034 records without duplicates; **Fig 1**). After title and abstract screening, 75 full-text articles were considered for inclusion. Nineteen individual studies published in 20 papers, comprising a total of 2492 participants, met the inclusion criteria for the systematic review. Overall, a wide range of protein intakes in low-protein or very low-protein diets from 0.29 to 0.9 g/kg/d were studied. The difference in protein intake between the treatment and control group ranged from 0.2 to 1.1 g/kg/d. The median number of study participants was 80 (range, 20–585), while the mean age of study participants was 51 years. These studies were continued for a median follow-up of 55 (range, 26–380) weeks. The median baseline eGFR was 33.5 (range, 14.4–96.3). Nine of the studies had keto acid supplementation as an intervention and the others did not. **Table 1** summarizes the characteristics of the included studies.

**Table 1:** Characteristics of included trials and patients.

**Study**	**Inclusion criteria** **(Defination of CKD)**	**Outcomes**	**Sample size**	**Follow-up time**	**Men (%)**	**Mean age**	**BMI**	**PI treatment**	**PI control**	**Mean eGFR**	**Mean proteinuria (g/d)**	**Funding Source**	**KA supplemental**	**Jadad^&^**
Ihle 1989	Scr (350–1000 umol/L) stable for 3 months	ESRD,GFR, ALB	64	78	67	37	NR	0.4^a^	≥0.75	14.42	NA	NR	no	3 (1, 1, 1)
Locatelli 1991	Ccr < 60 ml/min, Scr 133–619 umol/L	doubling of Scr, dialysis	456	104	NA	48	NR	0.718^b^	1	NA	NA	NR	no	4 (2, 1, 1)
Williams 1991	patient with deteriorating renal function, Scr > 130 mol/L	rate of Ccr decline, dialysis	95	82	NA	45	NR	0.71^c^	1.08	26.75*	3.15	Non-industry	no	4 (2,1, 1)
Zeller 1991	T1DM, proteinuria > 0.5 g/d, GFR 15–85% of normal	GFR, Ccr	35	152	60	34	NR	0.72 ^b^	1.08	47.37	3.62	NR	no	3 (1, 1, 1)
D'Amico 1994	Ccr 15–70 mL/min, stable for 3 months	halving of Ccr	128	117	61	54	NR	0.78 ^b^	1.03	33.13*	1.5	NR	no	3 (1, 1, 1)
MDRD study A 1994/2006	CKD patients, Scr > 106 umol/L, GFR 22–55 ml/min/ 1.73m^2^	GRF decline slop, RRT	585	114/380	61	51	NR	0.77 ^b^	1.11	38.6	0.2	NR	no	4 (2, 1, 1)
MDRD syudy B 1994/2009	CKD patients, Scr > 106 umol/L, GFR 13–24 ml/min/1.73m^2^	ESRD, death, GRF decline slop	255	114/380	59	51	NR	0.48 ^b^	0.73	18.5	0.7	NR	yes	4 (2, 1, 1)
Raal 1994	IDDM ≥10year, proteinuria ≥ 2 years	GFR, urinary protein	22	26	36	29	24.9	0.87 ^a^	2	58	2.03	NR	no	3 (1, 1, 1)
Iorio 2003	CRF patients, Ccr ≤ 25 mL/min/1.73 m2	Ccr, P, ALB	20	104	60	54	23.3	0.5 ^a^	0.79	16.35*	NA	NR	yes	3 (1, 1, 1)
Meloni DN 2004	DN, GFR 44.5±4.9 mL/min/1.73m^2^	GFR, BMI	80	52	47	54	24.8	0.86 ^c^	1.24	44.45	2.5	NR	no	2 (1, 1, 0)
Meloni non-DN 2004	non-DN, GFR 46.8±5.8 mL/min/1.73m^2^	GFR, BMI	89	52	51	62	26.8	0.67 ^c^	1.54	44.79	2	NR	no	2 (1, 1, 0)
Prakash 2004 (26)	predialytic CRF patients, Ccr 20–50 ml/min	GRF, nutritional parameters	34	39	50	54	25.2	0.3+Kas ^c^	0.6+placbo	28.34	NA	drug supplied by industry	yes	4 (1, 2, 1)
Mircescu 2007	nondiabetic patients with CKD, eGFR < 30 ml/min/1.73m^2^, proteinuria < 1 g/d, ALB > 35 g/L	eGFR, Scr, P, BMI, ALB, RRT	53	48	60	54	23.2	0.32^b^	0.59	17.02	0.61	NR	yes	2 (1, 0, 1)
**Study**	**Inclusion criteria**	**Outcomes**	**Sample size**	**Follow-up time**	**Men (%)**	**Mean age**	**BMI**	**PI treatment**	**PI control**	**Mean eGFR**	**Mean proteinuria (g/d)**	**Funding Source**	**KA supplemental**	**Jadad^&^**
Chen 2008	CAPD > 6 mon, ALB > 25 g/L	Scr, BMI, ALB,	78	52	58	67	23.7	0.8/0.8+ka^c^	1.2	NA	NA	NR	yes	4 (2, 1, 1)
Jiang 2009	stable PD patient	BMI, ALB, P, proteinuria	60	52	45	54	22	0.6–0.8/0.6–0.8+ka ^b^	1.0–1.2	NA	1.02	Non-industry and industry	yes	3 (1, 1, 1)
Koya 2009	T2DM > 5 years, patients had overt nephropathy, urinary protein 1–10 g/d, UAE > 200 ug/min, Scr < 176 umol/L	eGFR, doubling of Scr, ESRD, proteinuria ALB	112	260	59	57	NR	0.9^c^	1.1	62.3	1.15	Non-industry	no	3 (2, 0, 1)
Qiu 2012	predialytic T2DM patients, GFR < 60 ml/min/1.73m^2^, proteinuria > 0.3 g/d for 3 months ^2^,	proteinuria, GFR, BMI, ALB,	23	52	NA	62	NR	0.6+KA^c^	0.8	33.91	4.38	NR	yes	4 (2, 1, 1)
Zhang 2015	patients with incipient overt proteinuria, eGFR ≥ 60 ml/min/1.73m^2^	BMI, urinary protein, ALB, nutritional status	96	52	56	48	24.4	0.72 ^a^	1.09	96.34	3.86	Non-industry and industry	yes	3 (1, 1, 1)
Garneata 2016	eGFR < 30 ml/min//1.73m^2^, with stable renal function for at least 12 weeks proteinuria < 1 g/g Ucr, ALB > 3.5 g/dL	ESRD, GFR, proteinuria, ALB, BMI	207	65	61	54	23.4	0.29 ^c^	0.58	17.95	0.88	Fee offered by industry	yes	3 (1, 1, 1)

Abbreviations: ALB, albumin; BMI, body mass index; CAPD, continuous ambulatory peritoneal dialysis; Ccr, clearance of creatinine; CKD, chronic kidney disease; CRF, chronic renal failure; DN, diabetes nephropathy; eGFR, estimated glomerular filtration rate; ESRD, end stage renal disease; IDDM, insulin-dependent diabetes mellitus; KA, kato acid; NA, no available; NR, no reported; P, phosphorus; PI, protein intake; Scr, serum creatinine concentration; UAE, urine albumin excretion; Ucr, urine creatinine

Note: the units in the table: g/kg/day for protein intake; weeks for follow-up time; kg/m^2^ for BMI.

*Kidney function evaluated by Ccr

^&^The figures in bracket represent the detail Jadad score of each study (randomization, blinding, follow-up)

^a^ The calculation of protein intake was based on body weight

^b^ The calculation of protein intake was based on ideal body weight

^c^ The calculation of protein intake was not reported in article.

The methodological quality of the included trials was not high in general and varied substantially (**S1 and S2 Figs**). Specifically, 37% of the studies were at a low risk of bias in generation of the random sequence. The limit from implementation of a LPD meant that only 11% studies were at a low risk of bias for allocation concealment and 5% were at low risk of bias in blinding of participants and investigators to allocated treatment; 26% of the trials reported blinded outcome assessment. Incomplete outcome reporting was assessed as low risk in 79% of the studies, and selective outcome reporting was low risk in 63%. With respect to conflicts of interest, 5% of the RCTs were funded by the pharmaceutical industry and 16% reported author-industry financial relationships. Over all, seven trials had a Jadad scale of 4, nine trials of 3, and others scored 2.

### Effect of a low protein diet on kidney outcomes

**Fig 2:**
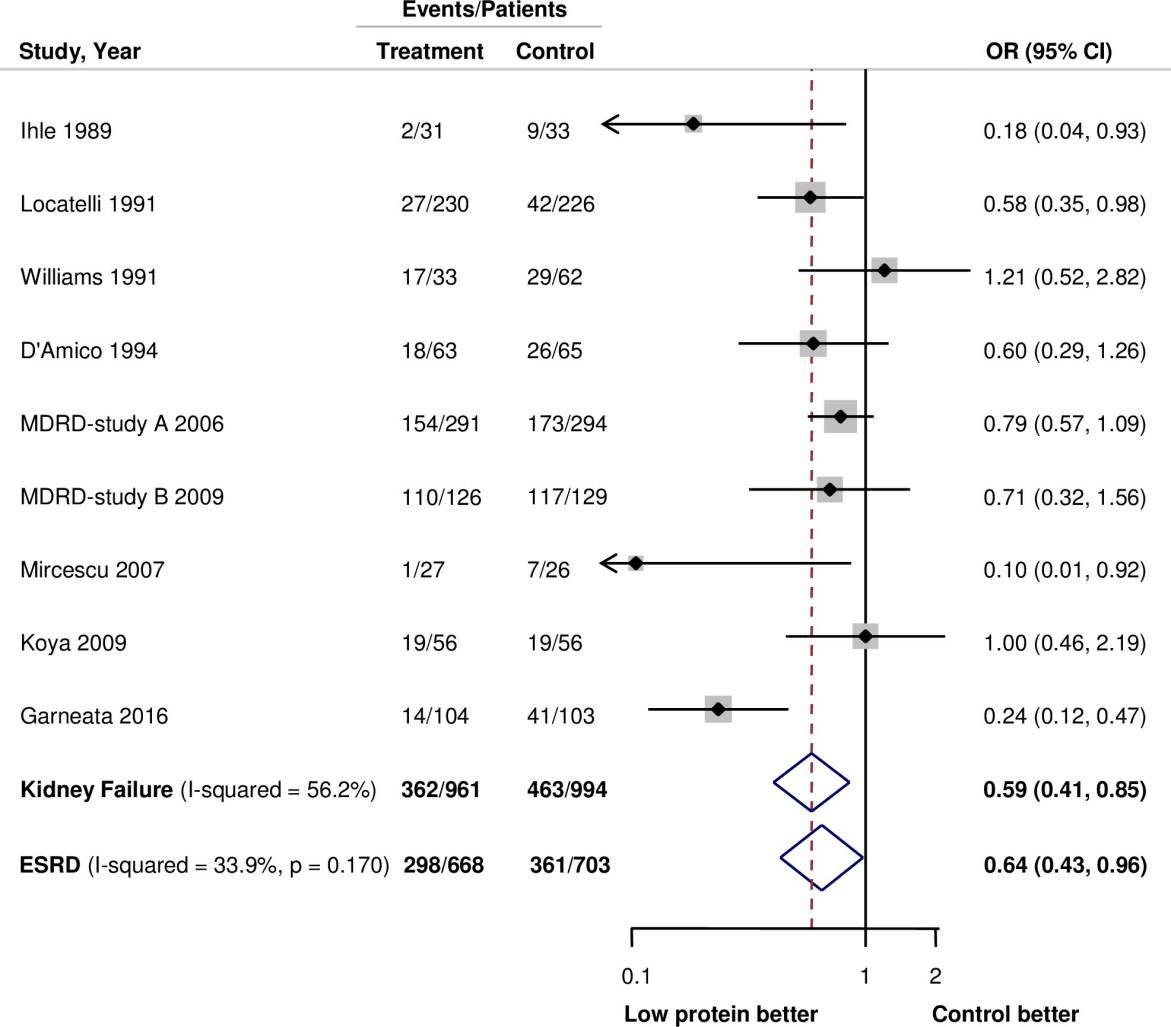
Forest plot for kidney failure events and ESRD. Abbreviations: CI, confidence interval; OR, odds ratio; ESRD, end-stage renal disease.

Kidney failure events, defined as a more than 25% decrease in the eGFR, doubling of serum creatinine, or ESRD, were reported in nine trials, including 1955 participants and 825 events. [13,14,24–30] Overall, lower protein intake reduced the risk of kidney failure events (OR: 0.59, 95% CI: 0.41 to 0.85) compared with control groups, with evidence of mild heterogeneity in the size of the effect across the included studies (*I*^*2*^ = 56%, *P* for heterogeneity = 0.02; **Fig 2**). When only ESRD among kidney failure events was considered (7 trials, 1371 participants, and 659 events)[13,14,24–26,28,30], the result did not changed (OR: 0.64, 95% CI: 0.43 to 0.96; *I*^*2*^ = 33.9%, *P* for heterogeneity = 0.17, **Fig 2**). Subgroup analysis was done for kidney failure events according to baseline characteristics (**Table 2**). The effect sizes were greater in trials that prescribed a lower protein intake in the protein restriction group (*P* for heterogeneity = 0.04). It also shows a tendency that a lower baseline mean proteinuria or eGFR was significantly associated with significant benefit in contrast to those with higher eGFR or proteinuria. On univariate meta-regression, there was a trend for low-protein diet to produce the greatest benefit when protein intake was restricted the most (**S3 Fig**, *P* = 0.01).

**Table 2:** Subgroup analysis of kidney failure events by outcome.

outcome	subgroup	No. of trials	Sample size	Statistic (OR/MD) (95%CI)	*P* value for statistic	*I*^2^ value	*P* value for heterogeneity test
**Kidney failure events**	**1. Mean eGFR (mL/min)**						
	< 30	5	674	0.41 (0.18, 0.94)	0.04	68.8%	0.2
	30–60	2	713	0.75 (0.56, 1.01)	0.06	0.0%	
	> 60	1	112	1.00 (0.46, 2.19)	1.0	-	
	**2. Mean proteinuria (g/day)**						
	< 1	4	1100	0.45 (0.21, 0.96)	0.04	75.8%	0.2
	1–3	2	240	0.76 (0.45, 1.31)	0.3	0.0%	
	> 3	1	95	1.21 (0.52, 2.82)	0.7	-	
	**3. Protein intake of experimental group (g/kg/day)**						
	< 0.6	4	579	0.30 (0.14, 0.67)	0.003	50.4%	0.04
	0.6–0.8	4	1264	0.74 (0.58, 0.95)	0.02	0.0%	
	> 0.8	1	112	1.00 (0.46, 2.19)	1.0	-	
	**4. DN or not**						
	Non-DN	5	908	0.37 (0.22, 0.65)	0.001	48.2%	0.2
	DN	1	112	1.00 (0.46, 2.19)	1.0	-	
	**5. Mean age (years)**						
	<51	3	615	0.62 (0.29, 1.36)	0.2	56.2%	0.8
	≥51	6	1340	0.56 (0.34, 0.92)	0.02	63.5%	

*Note*: The follow-up time of these nine studies included were more than 12 months.

**Fig 3:**
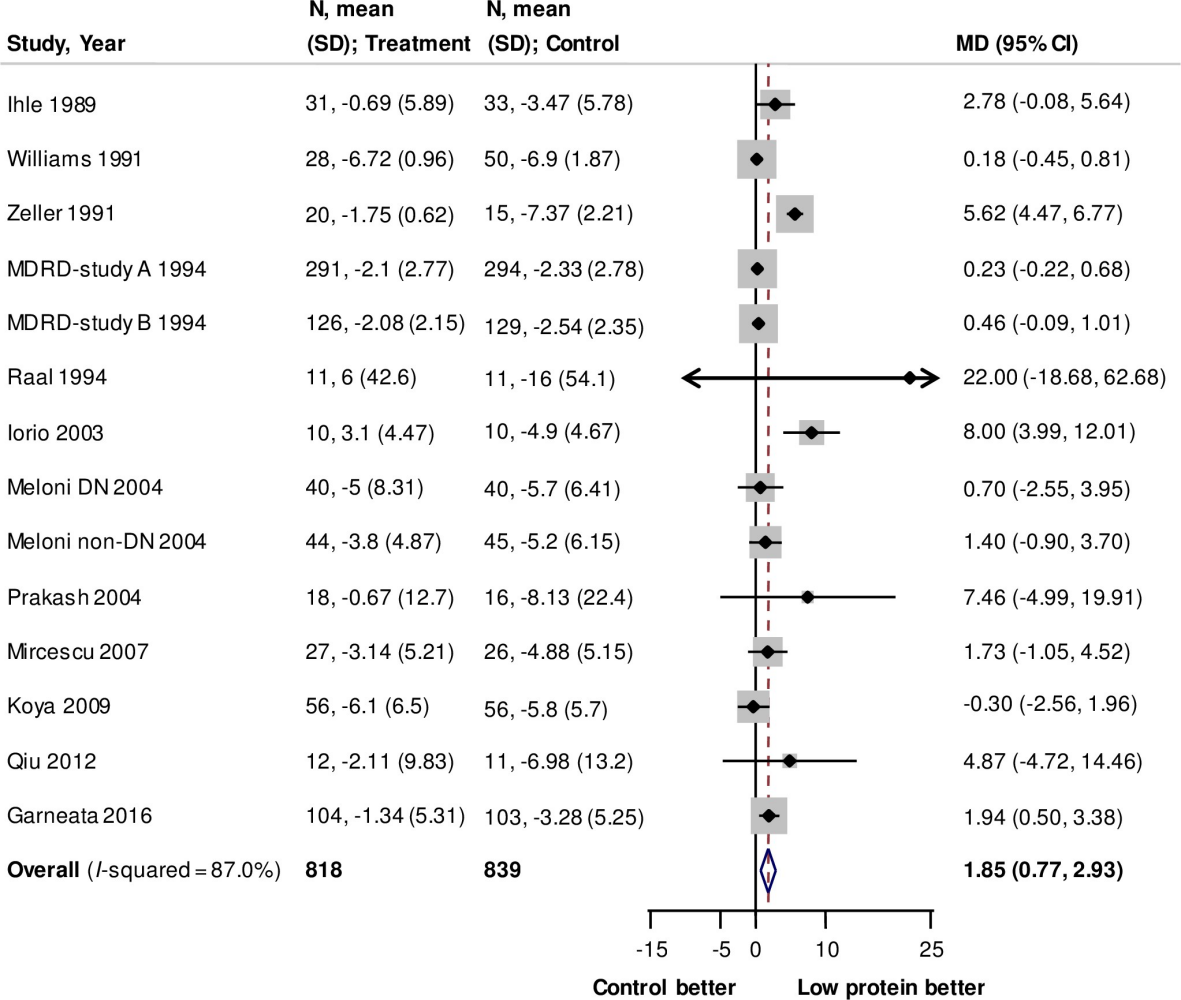
Forest plot for rate of change in eGFR. Positive values in difference of change represent slower decline for eGFR in treatment group than in control group. Abbreviations: CI, confidence interval; MD, mean difference; N, number of participants; SD, standard deviation.

Fourteen trials (1657 participants) reported data about the effects of low protein diet on the rate of change in eGFR. [8,13,25,26,28,30–36] Low protein intake slowed the rate of GFR decline by 1.85 ml/min/1.73 m^2^/year (95% CI: 0.77 to 2.93, *P* = 0.001, **Fig 3**) compared with that in the control groups. There was evidence of significant heterogeneity for the effects across the included studies (*I*^*2*^ = 87%). Subgroup analysis showed that there was heterogeneity for the effects of different baseline proteinuria (*P* = 0.02, **S1 Table**).

**Fig 4:**
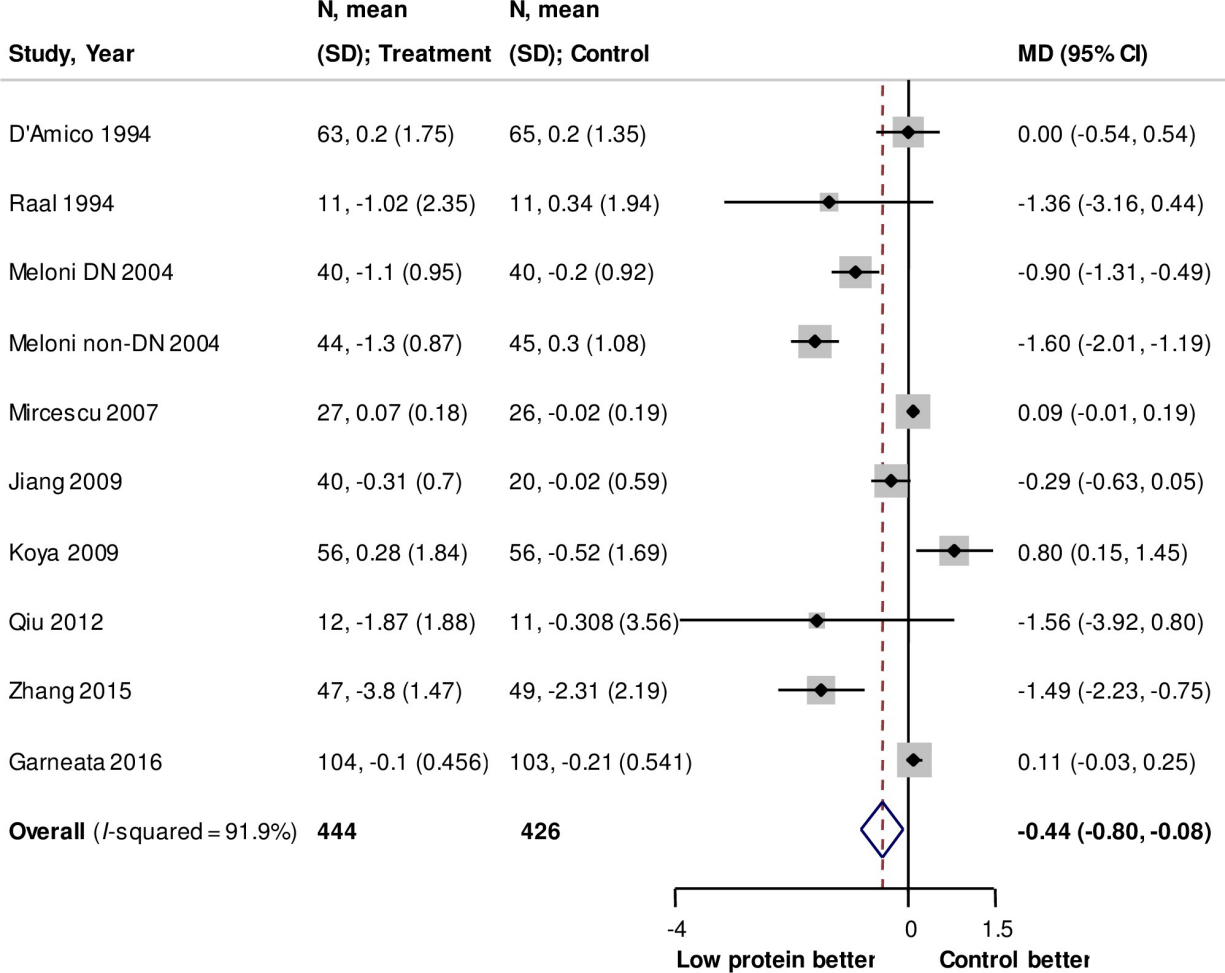
Forest plot for change in proteinuria. Negative values in difference of change represent greater decreases for proteinuria in low protein diet group than in control group. Abbreviations: CI, confidence interval; MD, mean difference; N, number of participants; PCR, protein to creatinine ratio; SD, standard deviation.

The effect of low protein diet on the change in proteinuria was available in ten trials with 870 participants. [13,25,29–31,33,34,37,38] All the data were extracted from urinary protein excretion with a simple unit conversion. Compared with the control groups, a low-protein diet reduced urinary protein excretion by 0.44 g/day (95% CI: −0.80 to −0.08, *P* = 0.02, **Fig 4**) with a substantial heterogeneity among the trials (*I*^2^ = 91.9%). There was no statistical heterogeneity for proteinuria in the subgroup analyses according to the prespecified characteristics (**S1 Table**). However, the change was not significant in dialysis patients (MD: −0.29, 95% CI: −0.63 to 0.05).

### Effect of a low protein diet on all-cause death

The effect of protein restriction on all-cause deaths was reported in five studies, including 1503 participants and 221 events. [14,24,27,28,30] Low protein diet was of little value in reducing the mortality of CKD patients compared with the control groups (OR: 1.17, 95% CI: 0.67 to 2.06, *P* = 0.6, **S4 Fig**) with no evidence of heterogeneity across these five trials (*I*^2^ = 43.1%, *P* for heterogeneity = 0.1).

### Effect of a low protein diet on the phosphorus concentration

Nine studies with 618 patients reported changes in the phosphorus concentration; [13,25,26,31,33–35,37] the pooled results showed that the MD in the change of phosphorus was statistically significant (−0.37, 95% CI: −0.50 to −0.24, *P* < 0.01) compared with the control groups (**S5 Fig**), with substantial heterogeneity (*I*^2^ = 75.6%). And the change was statistically significant both in dialysis (MD: −0.38, 95% CI: −0.60 to −0.16) and non-dialysis patients (MD: −0.37, 95% CI: −0.52 to −0.22).

### Effect of a low-protein diet on nutrient parameters

Twelve trials with a total of 1506 participants reported data on the change in Albumin, [13,25,31,33–37,39,40] and eight studies with 697 participants on the change in BMI. [13,25,34,36–38,40] The difference of the change in Albumin between two groups was not significant (MD: 0.23, 95% CI: −0.51 to 0.97, *I*^2^ = 92.4%, **S6 Fig**), both in dialysis and non-dialysis patients. But difference in the change in BMI had a slightly decrease compared with the control group (MD: −0.61, 95% CI: −1.05 to −0.17, *I*^2^ = 0%, **S7 Fig**), while the decrease was not significant in dialysis patients (MD: 0.13, 95% CI: −1.33 to 1.60).

### Sensitivity analysis

As shown in **S2 Table**, almost all the results did not substantially vary according to the prespecified characteristics in the sensitivity analysis, including omitting studies with follow-up of less than 12 months, omitting studies with a sample size of less than 50 participants, omitting studies with a Jadad score less than 3, and using different random-effects estimation methods. The most notable exceptions were that the effect of dietary protein restriction on proteinuria became insignificant when different statistical methods were used and three trials with low Jadad scores were removed. We noticed that the actual protein intake was greater than 0.8g/kg/day in three included studies, [30,33,34] which was considered as normal given. And two of he included studies were conducted in peritoneal dialysis patients. [37, 40] The post-hoc sensitivity analysis therefore was conducted excluding the two kinds of studies separately, and he results didn’t be changed (**S3 Table**).

## Discussion

Our systematic review and meta-analysis of 19 RCTs with 2492 participants showed that dietary protein restriction was associated with the risk reduction of kidney failure events. Significant benefits could also be achieved when only ESRD events were considered. These beneficial effects were consistent across major baseline characteristics and different follow-up times compared with the control group. Reduced dietary protein intake retards the rate of decline in GFR of 1.85 ml/min/1.73 m^2^/year, produces a decline in the proteinuria of 0.44 g/day, and was associated with a significant reduction of phosphorus concentration and a mild fall in BMI. However, summary estimates for the effects of dietary protein restriction on all-caused death were uncertain, and the difference of the change in albumin between the two groups also was not significant. Therefore, there was no suggestion that these adverse effects would outweigh the benefits of dietary protein restriction in CKD patients.

The current study represents a comprehensive summary of the benefits and risks of dietary protein restriction on different CKD participants with a wide range of eGFR (14–96), including both diabetic and non-diabetic CKD. As shown in **Table 3**, several systematic reviews and meta-analyses have pooled the available trials data in recent years. Fouque et al. confirmed a 32% risk reduction of the development of ESRD by protein restriction among non-diabetic patients with CKD.[7] When compared with this largest review published in 2009, 4 studies with 718 participants were excluded because of the different selection criteria, such as ages, follow-up time or the difference in protein intake and the reported outcomes. Kasiske et al. showed a moderate but significant protection by low protein diets (0.53 mL/min/y less loss in rate of eGFR for restricted protein intakes than for higher protein intakes). [41] These results were consistent with our study. The conflicting results were mainly associated with subjects with diabetic renal disease. Pedrini’s and Nezu’s study share a positive effect of low protein diet in slowing the progression of disease for patients with diabetic nephropathy, [6, 42] while the results from the subgroup analysis in our study is negative without heterogeneity between subgroup, including kidney failure events, eGFR and proteinuria (**Table 2 and S1 Table**). We assumed that the inconsistencies were related to the different included criterias. Only one RCT with 112 patients with DN reported kidney failure events in our study while Pedrini’s study included the nonrandomized crossover study and Nezu’s study did not evaluate the effects from hard endpoint. The results need to be confirmed by further study. Compared with the previous reviews, our study included only RCTs, and increased the numbers of participants available for analysis by 27% by including the latest published RCTs. [13,38]

**Table 3:** Summary of other meta-analysis.

Study, Year	Characteristics of patients include	Study type of trials included	Number of trials/patients	Outcome	Effective size	Conclusion
Fouque, 1992	Chronic renal insufficiency	RCT	6/890	Renal death	OR: 0.54 (0.37, 0.79)	LPD delay the onset of end stage renal death.
Pedrini, 1996	DN (T1DM)	RCT and nonrandomized crossover study	5/108	Change in UAE and decline in GFR/Ccr	RR: 0.56 (0.40, 0.77)	LPD effectively slows the progression of both diabetic and nondiabetic renal diseases
	Non-DN	RCT	5/1413	Renal failure or death	RR: 0.67 (0.50, 0.89)	
Kasiske, 1998	Nondiabetic and diabetic patients	RCT	13/1919	Change in GFR	WMD: 0.53 (0.08, 0.98)	Although LPD retards the rate of renal function decline, the magnitude of this effect is relatively weak.
Fouque, 2000	Non-DN	RCT	7/1494	Renal death	OR: 0.61 (0.46, 0.83)	LPD reduces the occurrence of renal death
Pan, 2008	DN	RCT	8/519	Change in GFR	WMD: 0.5 (-1.43, 2.42)	LPD did not significantly improve the renal function in patients with either types 1 or 2 DN.
				albumin	WMD: -1.18 (-1.33, 1.03)	
				HbA1C	WMD: -0.31 (-0.53, 0.09)	
				UPE/UAE	SMD: -0.69 (-1.14, -0.23)	
Fouque, 2009	Non-DN	RCT	10/2000	Renal death	RR: 0.68 (0.55, 0.84)	LPD reduces the occurrence of renal death
Nezu, 2013	DN	RCT	13/779	Change in GFR	WMD: 5.82 (2.3, 9.33)	LPD was significantly associated with improvement of DN.
				Proteinuria	SMD: -0.14 (-0.74, 0.46)	
				HbA1C	WMD: -0.26 (-0.35, -0.18)	
				albumin	WMD: -0.18 (-0.53, 0.17)	
Rughooputh, 2015	DN and non-DN	RCT	15/1965	Change in GFR	WMD: -0.95 (-1.79, -0.11)	LPD slows CKD progression in non-diabetic and in T1DM patients, but not in T2DM patients
Jiang, 2016	CKD	RCT, crossover and non-RCT design	9/410	GFR	WMD: -3.53 (-5.24, -1.82)	sLPD/sVLPD could delay the progression of CKD effectively without causing malnutrition.
				albumin	WMD: -0.95 (-2.62, 0.27)	
				phosphorus	WMD: -0.2 (-0.29, -0.11)	

Abbreviations: CKD, chronic kidney disease; DN, diabetes nephropathy; GFR, glomerular filtration rate; LPD, low protein diet; OR, odds ratio; RCT, randomized controlled trial; RR, relative risk; SMD, standard mean difference; WMD, weighted mean difference.

The largest RCT to date was the MDRD Study. [8] There was no difference in GFR decline between groups in Study A (GFR: 25 to 55 ml/min) and in Study B (GFR: 13 to 24 ml/min). Although there was a somewhat faster decline in GFR in the LPD group compared with the VLPD-KA group, this was not significant. However, secondary analysis of the MDRD study showed that each 0.2 g/kg/day decrease in protein intake was associated with a small amelioration in GFR decline over time (1.15 ml/min/1.73 m2/year), and with a 50% reduced risk of renal failure or death. [43] However, the relatively short period of the MDRD study and the unusually large proportion of polycystic kidney patients who often have very slow CKD progression might have reduced the study power.

It is our contention that dietary protein restriction may reduce the risk of kidney failure events, retard the rate of decline in eGFR, and decrease proteinuria in all cases of CKD. However, this study might be not the final answer for the question of LPD and progression of kidney disease. Several research questions arise from this work, perhaps the most important of which is how best to apply it in clinical practice. Many clinicians are concerned about worsening nutritional status and hence are reluctant to prescribe LPD. This is indeed true for patients with advanced CKD in whom there is spontaneous decrease in calorie and protein intake. In these cases, the absolute harm of treatment might uncomfortably surpass the absolute benefits. The amount of data available overall, and particularly that for persons with different CKD stages and types, was limited; as a result, insufficient power may explain some of the negative findings in our subgroup analysis and highlights the need for trials of LPD specifically targeting this different population. Furthermore, side effects and quality-of-life data were incompletely and inconsistently collected across the contributing studies, which made the interpretation and application of these findings challenging. The concern about malnutrition is the reason most clinicians do not advocate LPD despite the modest benefit. Further prospective study with malnutrition as an endpoint was needed to address the issue.

Second, adherence to LPD is a key element to gain its renoprotective effect. In all the included studies, compliance with a low LPD was poor. Given the high frequency of poor adherence, there has been criticism of the role of LPD in the real world clinical practice. Good patient-physician communication, self-monitoring of protein intake, and periodic feedback by the dietitian strengthens adherence through improved recognition of the importance of diet. [44]

Third, in the subgroup and meta-regression analysis, we observed a clear linear association between restricted dietary protein intake and the risk of kidney failure events, which partly explained the sources of heterogeneity in the analysis of the primary outcome. A recent RCT demonstrated that a vegetarian very low protein diet (VLPD, 0.3 g/kg/day) supplemented with ketoanalogs, compared with conventional LPD (0.6 g/kg/day), mitigated kidney function decline and reduced the number of patients requiring RRT. [13] Indeed, even a 0.1–0.2 g/kg/day reduction in protein intake from baseline appears to result in a significant effect on the preservation of kidney function. [45] There was no convincing or conclusive evidence for the optimal level of protein intake, especially when prescribed in different patients. [9]

Fourth, our study found no significant change in serum albumin levels or risk of all-caused death. A mild fall in body mass index in patients randomly assigned to a supplemented VLPD or an LPD might reflect inadequate attention to patients’ energy intake. Given that adequate calorie intake (30–35 kcal/kg/day) is needed to avoid protein catabolism and malnutrition under protein restriction of 0.6 g/kg/day or less, [46,47] uremic anorexia raises a question about the safety of LPD, which otherwise might induce malnutrition and aggravate protein-energy wasting (PEW) among patients with CKD. [9,48] Lower eGFR is associated with increased mortality risk both in general and kidney disease population cohorts [49–51]. However, our study showed that the significant benefits of LPD in the rate of eGFR and kidney failure events but did not in all-cause death. We assumed that the duration of the 5 studies reported all-cause death are too short to likely show a significant difference either positive or negative. In addition, the potential risks of malnutrition and PEW posed by LPD and the appearance of only late started dialysis not indeed benefits from LPD might also be the interpretations of the confliction.

The primary results of our study with beneficial effects should be explained and applied with caution. The protein intake target range should be individualized, and adequate calorie intake, close dietary counseling, and nutritional monitoring should be performed in consideration of the potential causing malnutrition. Our results also showed lower serum phosphorus levels in patients prescribed LPD, because dietary protein, especially animal protein, is a major source of phosphorus. The kidney benefit of a low protein diet might, at least in part, be related to its low phosphorus content, [52,53] as a decreased phosphorus burden can slow the progression of CKD and improve other outcomes in individuals with nondialysis-dependent CKD. [54,55]

The study does have some potential limitations. In general, evidence is of low or very-low quality, with suboptimal quality of the contributing studies and clinical heterogeneity, including considerable variation in trial duration, baseline kidney function, cause of CKD, and the definitions of LDP. The small sample size and low number of events in all-causes death, comprising only 5 trials with 221 events, might introduce a risk of false-negative results because of low statistical power, and the potential risk of malnutrition may confuse the result. Therefore, these results should be interpreted with caution; a meta-analysis does not eliminate the need for large, multicenter, randomized controlled trials, especially those with malnutrition as an endpoint. Second, individual participant data is lacking, which would have allowed a more reliable assessment of the treatment effects in different patient groups. Third, the existence of statistical heterogeneity in the several outcome analyses might have affected our results, although we attempted to address these through the use of random-effects models and subgroup analysis.

In conclusion, dietary protein restriction may delay the progression of CKD, including risk reduction of kidney failure events, decline in the rate of eGFR and proteinuria levels as compared with higher or unrestricted protein intake. Although the benefits from LPD in patients with CKD exist, individual prescription, careful follow-up by a physician in conjunction with a skilled dietitian to assess protein and energy intake, and nutritional status, should be considered. The optimal protein intake for different participants remains unanswered, and further studies targeting different CKD populations are warranted to confirm and integrate these results.

## Supporting information

S1 Checklist PRISMA 2009 checklist. (DOC)

S1 Item Study protocol (including search strategies). (DOCX)

S2 Item Risk of bias for the outcome. (DOCX)

S1 Fig Risk of bias graph. (TIF)

S2 Fig Risk of bias summary. (TIF)

S3 Fig Influence of different protein intake on kidney failure events. (TIF)

S4 Fig Forest plot for all cause death. Abbreviations: CI, Confidence interval.
(TIF)

S5 Fig Forest plot for change in phosphorus. Abbreviations: CI, Confidence interval; MD, mean difference.
(TIF)

S6 Fig Forest plot for change in albumin. Abbreviations: CI, Confidence interval; MD, mean difference.
(TIF)

S7 Fig Forest plot for change in BMI. Abbreviations: CI, Confidence interval; MD, mean difference.
(TIF)

S1 Table Subgroup analysis of kidney function by outcome. (DOCX)

S2 Table Sensitivity analyses of kidney function. *Note*: * all the studies included had relatively large sample size more than 50. Kidney failure events was defined as doubling of serum creatinine level or 50% decline in estimated GFR or end-stage renal disease. GFRs expressed in mL/min/1.73m^2^. Abbreviations: CI, confidence intervals; D-L; DerSimonian-Laird; EB, empirical Bayes; eGFR, estimated glomerular filtration rate; MD, mean difference; n, number of patients; No. number of trials; OR, odds ratio; REML, restricted maximum likelihood;.
(DOCX)

S3 Table Post-hoc sensitivity analyses. *Note*: sensitivity analysis A, Results with exclusion of studies with protein intake greater than 0.8 g/kg /day; sensitivity analysis B, Results with exclusion of dialysis patients.
(DOCX)
