# Supplementary material for: Effect of diet protein restriction on progression of chronic kidney disease: A systematic review and meta-analysis
Source: PLoS One. 2018 Nov 7;13(11):e0206134. doi: 10.1371/journal.pone.0206134 (PMC6221301; doi:10.1371/journal.pone.0206134)
Supplement: S1 Item — (DOCX) [file pone.0206134.s002.docx]

**Study protocol**

**Effect of Diet Protein Restriction on Progression of Chronic Kidney Disease, a Systematic Review and Meta-analysis**

Bingjuan Yan, Xiaole Su, Boyang Xu, Xi Qiao, and Lihua Wang

Renal Division, Shanxi Medical University Second Hospital

**Review question**

Dietary protein has long been thought to play an important role in the progression of chronic kidney disease (CKD), but what effect dietary protein restriction has on the rate of decline in kidney function is unclear. We will assess the effects of different levels of dietary protein restriction on the rate of decline in eGFR and explore the effects of differences in underlying cause of CKD, level of dietary protein and phosphorous on kidney function.

# Searches

**Data sources**

All relevant randomized controlled trails (RCTs) will be roundly searched in following electronic databases without language restriction: Ovid MEDLINE SP (from 1946 through Mar week 1 2016), EMBASE (from 1966 through Mar week 1 2016), and the Cochrane Central Register of Controlled Trails (no date restriction). Other resources include a manual search of reference list of review articles and relevant studies, and biomedical companies contacted. A search in [www.clinicaltrials.gov](http://www.clinicaltrials.gov) will be used to identify the ongoing but unpublished studies about this field.

**Study selection**

**Participants/population:** Adult patients with CKD (as defined by KDIGO 2012 Clinical Practice Guideline for the Evaluation and Management of CKD) will be included without restrictions on gender or race.

**Intervention(s), exposure(s):** Participants treated with a lower protein intake (0.6-0.8g/kg/day) or a very-low protein diet (0.3g/kg/day).

**Comparator(s)/control:** Difference in protein intake between control and treated groups of at least 0.2g protein/kg/day.

**Types of study to be included initially**

We will include RCTs that compared different levels of protein intake (mentioned in Intervention and Comparator) for adult patients with CKD or RCTs of broader populations for which data for participants with CKD could be disaggregated.

**Primary outcomes**

1) Rate of change in eGFR: The difference from baseline in eGFR divided by the number of years between creatinine measurements (ml/min/1.73m2 per year).

2) Kidney failure events: If the data allow, it will include more than 25% or 50% decrease in the estimated GFR (eGFR)*, doubling of serum creatinine, end stage renal disease.

*The estimated GFR included creatinine clearance rate and eGFR by MDRD or CG formula, creatinine clearance was used interchangeably with GFR to assess all outcomes.

**Secondary outcomes**

If the data allow，

1) Nutrition state reflected by serum albumin, hematocrystallin, body weight or BMI;

2) Change in proteinuria or albuminuria, including urinary protein excretion (UPE), urinary albumin excretion (UAE), protein to creatinine (PCR) and albumin to creatinine ratio (ACR);

3) Change in serum lipid level;

4) Change in serum phosphates;

5) Change in serum creatinine;

6) All-cause mortality.

# Data extraction, (selection and coding)

Data extraction will be carried out independently by two reviewers using standard data extraction forms. Disagreement will be resolved by consensus or by discussion with a third author. When detailed information that was needed for the analysis was not available, the original authors will be contacted to obtain the missing information through e-mail. Where multiple publications of one study exist, data will be extracted and grouped together as a single study with the most complete data.

**The extracted data will include:**

· study characteristics (design, method of randomization, and withdrawals/dropouts);

· baseline patient characteristics (age, sex, cause of renal disease, mean proteinuria or albuminuria, eGFR, serum creatinine concentrations; diabetic status, hypertension status, smoking status, visceral obesity, previous cardiovascular events);

· antiplatelet agents used; dose and route of administration; follow-up duration;

· outcomes: as listed in Primary outcomes and Secondary outcomes

# Risk of bias (quality) assessment

We will assess risk of bias for sequence generation, allocation concealment, blinding, selective reporting, incomplete outcome data and other sources of bias, and determined overall risk of bias based on predefined rules, utilizing the Cochrane Collaboration risk of bias tool. (reference: Higgins JP, Altman DG, et al. Cochrane Statistical Methods G. The Cochrane Collaboration's tool for assessing risk of bias in randomized trials. BMJ 2011;343:d5928.). Furthermore, the study quality will be quantified with Jadad scale. (reference: Jadad AR, Moore RA, Carroll D, et al. Assessing the quality of reports of randomized clinical trials: is blinding necessary? Control Clin Trials 1996;17:1– 12.)

# Strategy for data synthesis

In the bivariate outcome analysis, if the odds ratios (ORs) are unavailable in the original article, individual study ORs and 95% CIs will be calculated from event numbers and total population at risk extracted from each trial before data pooling. Mean differences (MD) will be used to pool the rate of change in eGFR and creatinine clearance. Standardized mean differences (SMD) will be used to pool continuous variable data from all studies that reported change in proteinuria or albuminuria (including UAE, UPE, ACR or PCR), and other available blood biochemical indexes. Because of the differences expected between studies, a random-effects model will be used to combine results. Statistical heterogeneity was quantified using the *I*^2^ statistic. Sensitivity analyses will be conducted to check the robustness of the effect of protein-restricted treatment by excluding of trials with relative small sample size, with follow year <1 year and with low Jadad scores. A two-sided *P*-value less than 0.05 was considered statistically significant and statistical analyses were performed using RevMan version 5.3 and STATA version 12.0.

# Analysis of subgroups or subsets

If the data allow, subgroup analysis will be performed by mean age, sexual, cause of kidney disease, baseline mean eGFR, serum creatinine, phosphates, mean proteinuria or albuminuria, follow-up duration and DM or no-DM participants.

**Search strategy**

**MEDLINE**

1. exp Kidney/ 318260
2. Kidney.mp. 707078
3. renal.mp. 564210
4. kidney disease.mp. or exp Kidney Diseases/ 452162
5. Renal Insufficiency.mp. or exp Renal Insufficiency/ 146401
6. (ESRF or ESKF or ESRD or ESKD).mp. [mp=title, abstract, original title, name of substance word, subject heading word, keyword heading word, protocol supplementary concept word, rare disease supplementary concept word, unique identifier] 13157
7. (CKF or CKD or CRF or CRD).mp. [mp=title, abstract, original title, name of substance word, subject heading word, keyword heading word, protocol supplementary concept word, rare disease supplementary concept word, unique identifier] 30551
8. Renal Replacement Therapy.mp. or exp Renal Replacement Therapy/ 180085
9. exp Peritoneal Dialysis/ or exp Dialysis/ or exp Renal Dialysis/ or exp Peritoneal Dialysis, Continuous Ambulatory/ 119984
10. (haemodialysis or hemodialysis).mp. [mp=title, abstract, original title, name of substance word, subject heading word, keyword heading word, protocol supplementary concept word, rare disease supplementary concept word, unique identifier] 64646
11. CAPD.mp. 6324
12. renal transplantation.mp. or exp Kidney Transplantation/ 86841
13. ((kidney or renal) adj graft*).mp. [mp=title, abstract, original title, name of substance word, subject heading word, keyword heading word, protocol supplementary concept word, rare disease supplementary concept word, unique identifier] 5735
14. 1 or 2 or 3 or 4 or 5 or 6 or 7 or 8 or 9 or 10 or 11 or 12 or 13 1015079
15. Protein Deficiency.mp. or exp Protein Deficiency/ 12694
16. protein restricted diet.mp. or Diet, Protein-Restricted/ 2472
17. low protein diet.mp 2851
18. very-low-protein diet.mp. 89
19. exp Dietary Proteins/ or protein intake. 86838
20. Essential Amino Acids.mp. or exp Amino Acids, Essential/ 197007
21. Keto Acids.mp. or exp Keto Acids/ 43143
22. 15 or 16 or 17 or 18 or 19 or 20 or 21 329340
23. randomized controlled trial.pt. 408342
24. controlled clinical trial.pt. 90181
25. randomized.ab. 337781
26. randomly.ab. 243590
27. trial.ti 146651
28. clinical trials as topic.sh. 175120
29. 23 or 24 or 25 or 26 or 27 or 28 963940
30. (animals not (humans and animals)).sh. 4161827
31. 29 not 30 891347
32. 14 and 22 and 31 1179

**EMBASE**

1. 'kidney'/exp OR kidney 1092302

2. renal   712443

3. 'kidney disease'/exp OR 'kidney disease' 772746

4. 'kidney failure'/exp OR 'kidney failure' 264686

5. 'renal insufficiency'/exp OR 'renal insufficiency' 269554

6. 'esrd'/exp OR esrd OR eskd OR esrf OR eskf 26684

7. crd OR ckd OR 'crf'/exp OR crf OR ckf 59044

8. 'renal replacement therapy'/exp OR 'renal replacement therapy' 153132

9. 'renal dialysis'/exp OR 'renal dialysis' 151589

10. 'dialysis'/exp OR 'dialysis' 170328

11. 'hemodialysis'/exp OR 'hemodialysis' OR 'haemodialysis'/exp OR 'haemodialysis' 118221

12. 'peritoneal dialysis'/exp OR 'peritoneal dialysis' 39324

13. 'capd'/exp OR 'capd' 13102

14. 'renal transplantation'/exp OR 'renal transplantation' 129245

15. 'renal graft'/exp OR 'renal graft' 29026

16. 'kidney'/exp OR kidney OR renal     OR 'kidney disease'/exp OR 'kidney disease' OR 'kidney failure'/exp OR 'kidney failure' OR 'renal insufficiency'/exp OR 'renal insufficiency' OR 'esrd'/exp OR esrd OR eskd OR esrf OR eskf OR crd OR ckd OR 'crf'/exp OR crf OR ckf OR 'renal replacement therapy'/exp OR 'renal replacement therapy' OR 'renal dialysis'/exp OR 'renal dialysis' OR 'dialysis'/exp OR 'dialysis' OR 'hemodialysis'/exp OR 'hemodialysis' OR 'haemodialysis'/exp OR 'haemodialysis' OR 'peritoneal dialysis'/exp OR 'peritoneal dialysis' OR 'capd'/exp OR 'capd' OR 'renal transplantation'/exp OR 'renal transplantation' OR 'renal graft'/exp OR 'renal graft' 1456546 17. 'low protein diet'/exp OR 'low protein diet' 7,679

18. 'protein deficiency'/exp OR 'protein deficiency' 29090

19. 'protein restricted diet'/exp OR 'protein restricted diet' 6085

20. 'protein restricted' AND ('diet'/exp OR diet) 776

21. 'low protein' AND ('diet'/exp OR diet) 5471

22. 'very low protein' AND ('diet'/exp OR diet) 151

23. 'protein intake'/exp OR 'protein intake' 36568

24. 'dietary protein'/exp OR 'dietary protein' 36859

25. 'essential amino acid'/exp OR 'essential amino acid' 264759

26. 'keto acids'/exp OR 'keto acids' 85694

27. 'ketoacid'/exp OR 'ketoacid' 85875

28. 'low protein diet'/exp OR 'low protein diet' OR 'protein deficiency'/exp OR 'protein deficiency' OR 'protein restricted diet'/exp OR 'protein restricted diet' OR ('protein restricted' AND ('diet'/exp OR diet)) OR ('low protein' AND ('diet'/exp OR diet)) OR ('very low protein' AND ('diet'/exp OR diet)) OR 'protein intake'/exp OR 'protein intake' OR 'dietary protein'/exp OR 'dietary protein' OR 'essential amino acid'/exp OR 'essential amino acid' OR 'keto acids'/exp OR 'keto acids' OR 'ketoacid'/exp OR 'ketoacid' 410246

29. 'randomized controlled trial'/exp OR 'randomized controlled trial' 490498

30. 'controlled clinical trial'/exp OR 'controlled clinical trial' 526707

31. random* 1216912

32. 'clinical trials'/exp OR 'clinical trials' 376620

33. trial:ti 199076

34. 'randomized controlled trial'/exp OR 'randomized controlled trial' OR 'controlled clinical trial'/exp OR 'controlled clinical trial' OR random* OR 'clinical trials'/exp OR 'clinical trials' OR trial:ti 1608582

35. 'human' 17542866

36. animal$ OR nonhuman 6 931921

37. 'human' AND (animal$ OR nonhuman) 1953578

38. animal$ OR nonhuman NOT ('human' AND (animal$ OR nonhuman)) 4978343

39. 'randomized controlled trial'/exp OR 'randomized controlled trial' OR 'controlled clinical trial'/exp OR 'controlled clinical trial' OR random* OR 'clinical trials'/exp OR 'clinical trials' OR trial:ti NOT (animal$ OR nonhuman NOT ('human' AND (animal$ OR nonhuman))) 1,464,821

40. 'kidney'/exp OR kidney OR renal OR 'kidney disease'/exp OR 'kidney disease' OR 'kidney failure'/exp OR 'kidney failure' OR 'renal insufficiency'/exp OR 'renal insufficiency' OR 'esrd'/exp OR esrd OR eskd OR esrf OR eskf OR crd OR ckd OR 'crf'/exp OR crf OR ckf OR 'renal replacement therapy'/exp OR 'renal replacement therapy' OR 'renal dialysis'/exp OR 'renal dialysis' OR 'dialysis'/exp OR 'dialysis' OR 'hemodialysis'/exp OR 'hemodialysis' OR 'haemodialysis'/exp OR 'haemodialysis' OR 'peritoneal dialysis'/exp OR 'peritoneal dialysis' OR 'capd'/exp OR 'capd' OR 'renal transplantation'/exp OR 'renal transplantation' OR 'renal graft'/exp OR 'renal graft' AND ('low protein diet'/exp OR 'low protein diet' OR 'protein deficiency'/exp OR 'protein deficiency' OR 'protein restricted diet'/exp OR 'protein restricted diet' OR ('protein restricted' AND ('diet'/exp OR diet)) OR ('low protein' AND ('diet'/exp OR diet)) OR ('very low protein' AND ('diet'/exp OR diet)) OR 'protein intake'/exp OR 'protein intake' OR 'dietary protein'/exp OR 'dietary protein' OR 'essential amino acid'/exp OR 'essential amino acid' OR 'keto acids'/exp OR 'keto acids' OR 'ketoacid'/exp OR 'ketoacid') AND ('randomized controlled trial'/exp OR 'randomized controlled trial' OR 'controlled clinical trial'/exp OR 'controlled clinical trial' OR random* OR 'clinical trials'/exp OR 'clinical trials' OR trial:ti) NOT (animal$ OR nonhuman NOT ('human' AND (animal$ OR nonhuman))) 1929

**CENTRAL**

#1 renal:ti,ab,kw (Word variations have been searched)  30382

#2 "kidney":ti,ab,kw (Word variations have been searched) 26689

#3 MeSH descriptor: [Kidney] explode all trees 3542

#4 MeSH descriptor: [Kidney Diseases] explode all trees 11146

#5 kidney diseases:ti,ab,kw (Word variations have been searched) 5300

#6 MeSH descriptor: [Renal Insufficiency] explode all trees 5613

#7 "kidney failure":ti,ab,kw (Word variations have been searched) 6767

#8 CKD or CKF or CRD or CRF: ti, ab, kw (Word variations have been searched ) 1955

#9 ESKD or ESKF or ESRD or ESRF: ti,ab,kw (Word variations have been searched) 894

#10 "renal replacement therapy":ti,ab,kw (Word variations have been searched) 830

#11 "dialysis":ti,ab,kw (Word variations have been searched) 8665

#12 MeSH descriptor: [Renal Dialysis] explode all trees 4754

#13 renal dialysis:ti,ab,kw (Word variations have been searched) 6831

#14 "hemodialysis" or haemodialysis:ti,ab,kw (Word variations have been searched) 6069

#15 "peritoneal dialysis":ti,ab,kw (Word variations have been searched) 1461

#16 CAPD:ti,ab,kw (Word variations have been searched) 558

#17 "kidney transplantation":ti,ab,kw (Word variations have been searched) 5780

#18 kidney graft:ti,ab,kw (Word variations have been searched) 4247

#19 MeSH descriptor: [Kidney Transplantation] explode all trees 3531

#20 #1 or #2 or #3 or #4 or #5 or #6 or #7 or #8 or #9 or #10 or #11 or #12 or #13 or #14 or #15 or #16 or #17 or #18 or #19 42393

#21 "low-protein diet":ti,ab,kw (Word variations have been searched) 294

#22 "low protein diet":ti,ab,kw (Word variations have been searched) 294

#23 protein deficiency:ti,ab,kw (Word variations have been searched) 1463

#24 protein restricted diet:ti,ab,kw (Word variations have been searched) 765

#25 protein-restricted diet:ti,ab,kw (Word variations have been searched) 252

#26 very-low-protein diet:ti,ab,kw (Word variations have been searched) 48

#27 protein intake:ti,ab,kw (Word variations have been searched) 5665

#28 "dietary protein":ti,ab,kw (Word variations have been searched) 2385

#29 essential amino acids:ti,ab,kw (Word variations have been searched) 537

#30 'keto acids' or ketoacids:ti,ab,kw (Word variations have been searched) 345

#31 #21 or #22 or #23 or #24 or #25 or #26 or #27 or #28 or #29 or #30 8227

#32 #20 and #31 1154
