# Supplementary material for: Effect of diet protein restriction on progression of chronic kidney disease: A systematic review and meta-analysis
Source: PLoS One. 2018 Nov 7;13(11):e0206134. doi: 10.1371/journal.pone.0206134 (PMC6221301; doi:10.1371/journal.pone.0206134)
Supplement: S2 Item — (DOCX) [file pone.0206134.s003.docx]

**Risk of bias for the outcome**

We assessed risk of bias for sequence generation, allocation concealment, blinding, selective reporting, incomplete outcome data and other sources of bias (sponsorship bias), and determined overall risk of bias based on predefined rules, utilizing the Cochrane Collaboration risk of bias tool.

**Cochrane collaboration’s tool for assessing risk of bias:**

**Sequence generation (Selection bias)**

• Low risk of bias, if randomization was generated by a computer, or a table of random numbers.

• High risk of bias, if method of randomization was inadequate (i.e. "quasi-randomized").

• Unclear risk of bias, if method of randomization was not described.

**Allocation concealment** **(Selection bias)**

• Low risk of bias, if the method of allocation involved a central independent unit or consecutively numbered sealed envelopes.

• High risk of bias, if allocation sequence was known to the investigators or conducted with an inadequate method.

• Unclear risk of bias, if the method of allocation concealment was not described.

**Blinding of participants and personnel (Performance bias)**

• Low risk of bias, if the study was of a double-blind design.

• High risk of bias, if the study was open-label.

• Unclear risk of bias, if there was insufficient information to determine whether the study was double-blind or open-label.

**Blinding of outcome assessment**

• Low risk of bias, no blinding of outcome assessment, but the review authors judge that the outcome measurement is not likely to be influenced by lack of blinding; blinding of outcome assessment ensured, and unlikely that the blinding could have been broken

• High risk of bias: No blinding of outcome assessment, and the outcome measurement is likely to be influenced by lack of blinding; blinding of outcome assessment, but likely that the blinding could have been broken, and the outcome measurement is likely to be influenced by lack of blinding
• Unclear: Insufficient information to permit judgement

**Selective outcome reporting (Detection bias)**

• Low risk of bias, if the specific outcome was reported adequately for all treatment arms.

• High risk of bias, if the specific outcome was reported with inadequate detail for the data to be included in a meta-analysis or if it was reported only for a subset of the randomized population.

• Unclear risk of bias, if there was insufficient information to assess whether the risk of bias of selective outcome reporting was present.

**Incomplete outcome data (Attrition bias)**

• Low risk of bias, if

1. attrition rate was balanced between treatment arms and relatively low (below 20%), and

2. reasons for discontinuation were described, and

3. an intention-to-treat analysis was performed, and

4. an appropriate method of imputation of missing outcome data was applied.

• High risk of bias, if

1. withdrawal rates were unbalanced between treatment arms or more than 20%, or

2. reasons for drop-outs were not clearly described, or

3. an inappropriate analysis was performed (i.e. per protocol analysis), or

4. an inappropriate imputation method (i.e. last observation carried forward method) was used to handle missing data.

• Unclear risk of bias, if it is not clear whether there were any drop-outs, or reasons for these withdrawals are not clear, or no method of imputation of missing data is mentioned.

**Other bias** **(Sponsor bias)**

• Low risk of bias, if the trial was not funded by a drug manufacturer.

• High risk of bias, if the trial was funded by a drug manufacturer.

• Unclear risk of bias, if the source of funding was unclear.
