# Supplementary figures and images for: Effect of diet protein restriction on progression of chronic kidney disease: A systematic review and meta-analysis
Source: PLoS One. 2018 Nov 7;13(11):e0206134. doi: 10.1371/journal.pone.0206134 (PMC6221301; doi:10.1371/journal.pone.0206134)

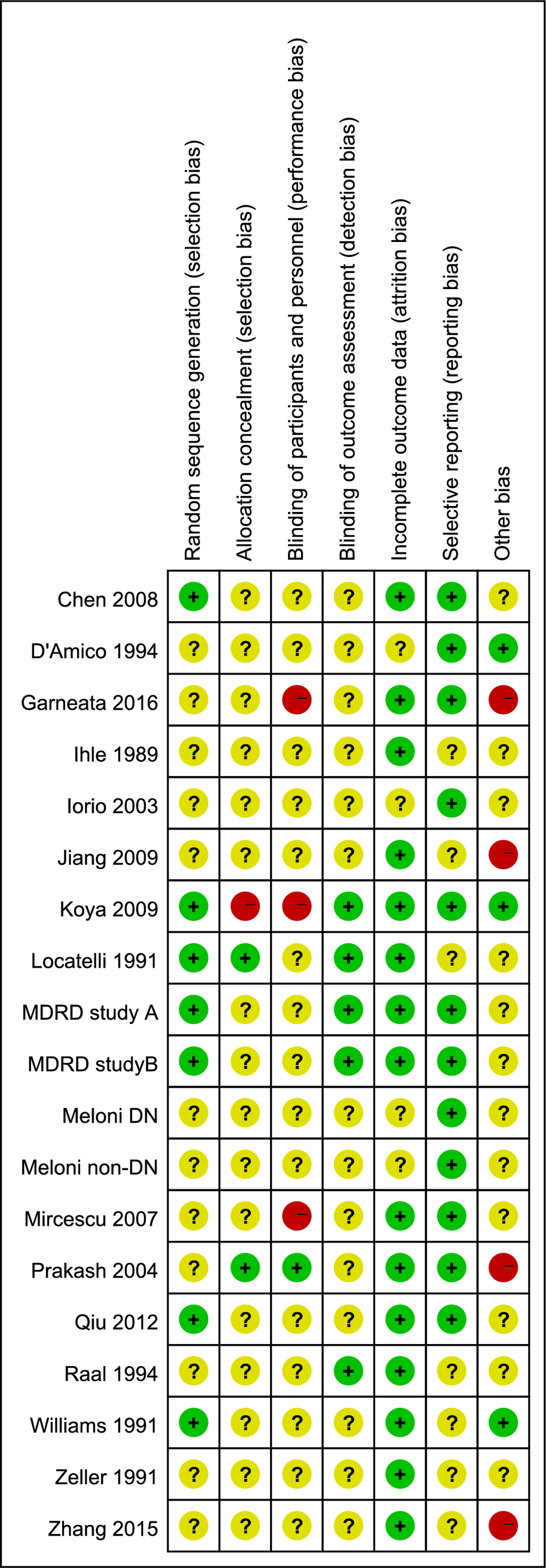

Supplement: S1 Fig — (TIF) [file pone.0206134.s004.tif]

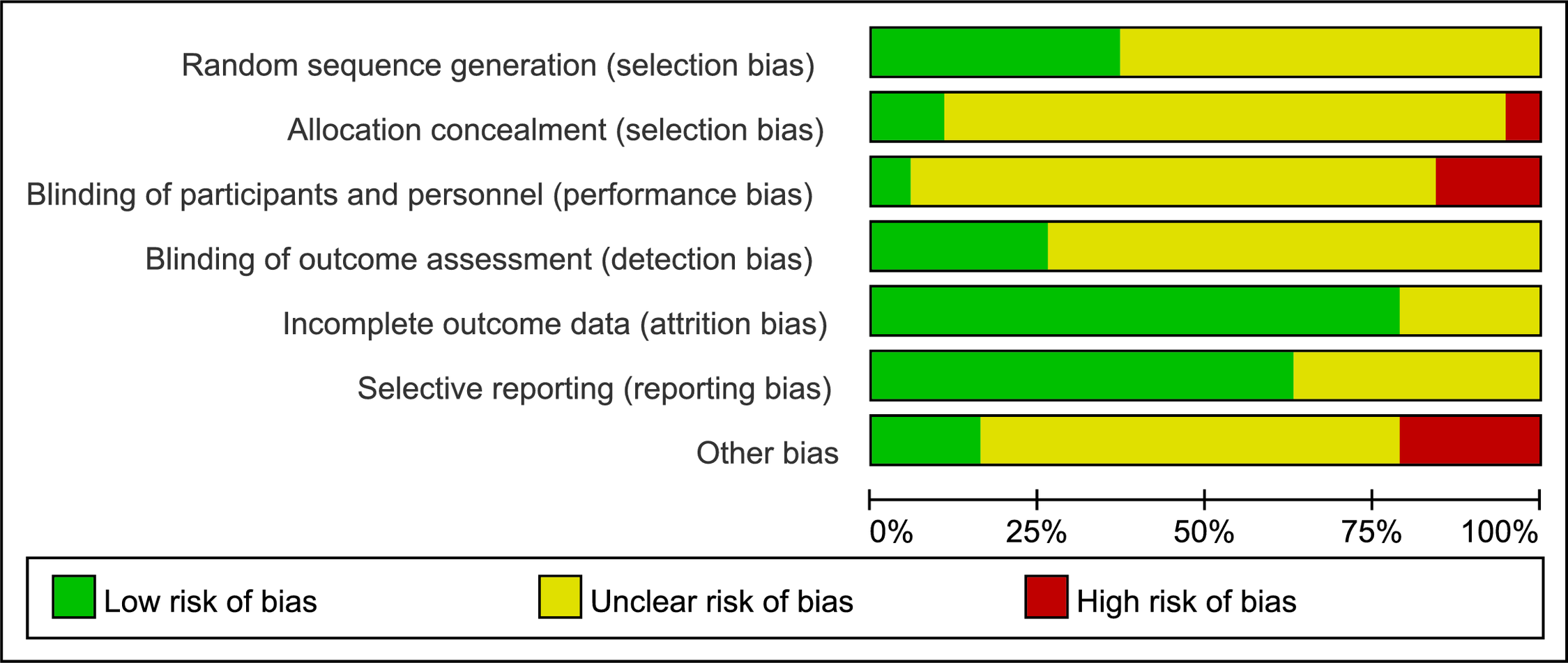

Supplement: S2 Fig — (TIF) [file pone.0206134.s005.tif]

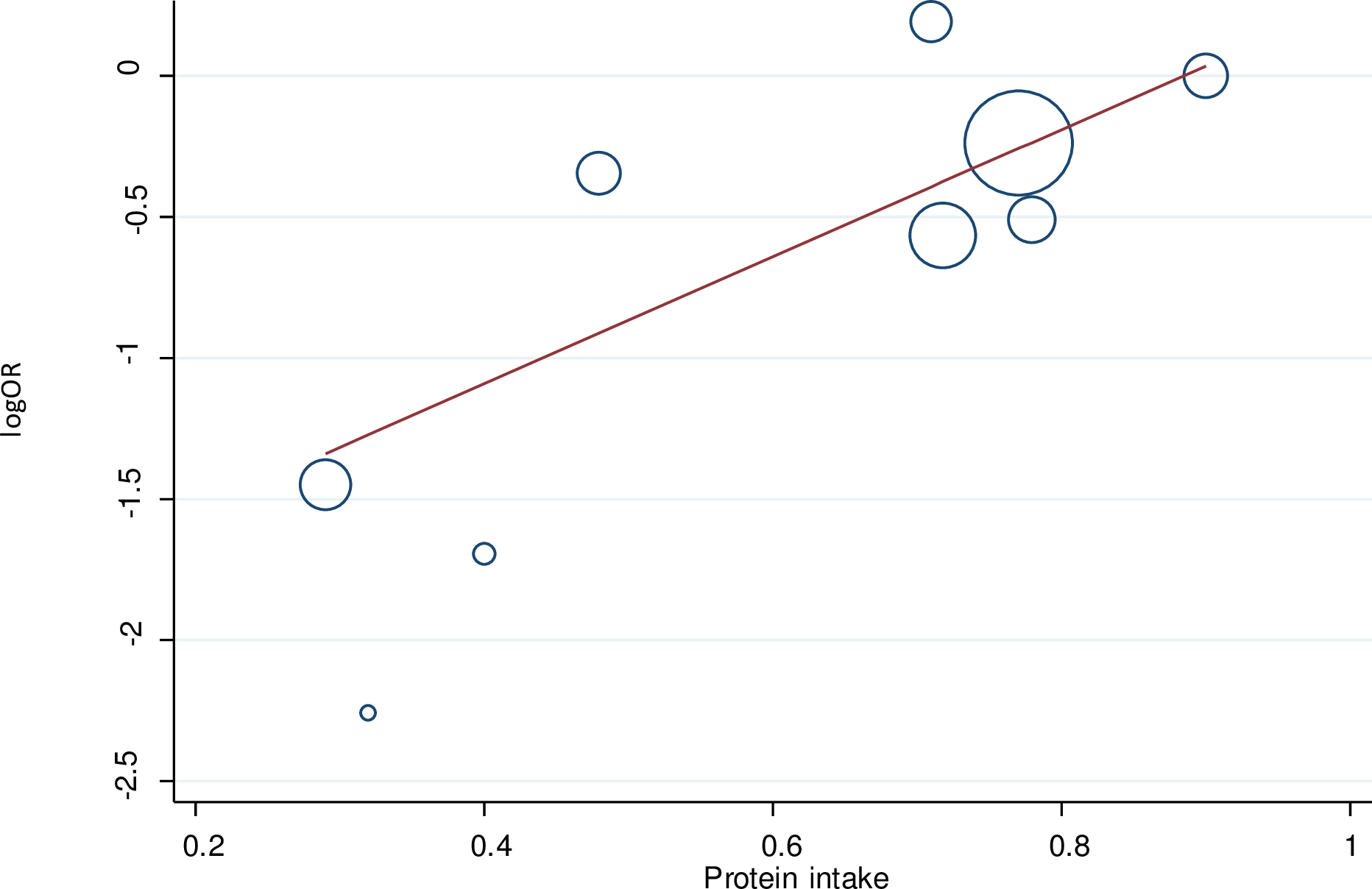

Supplement: S3 Fig — (TIF) [file pone.0206134.s006.tif]

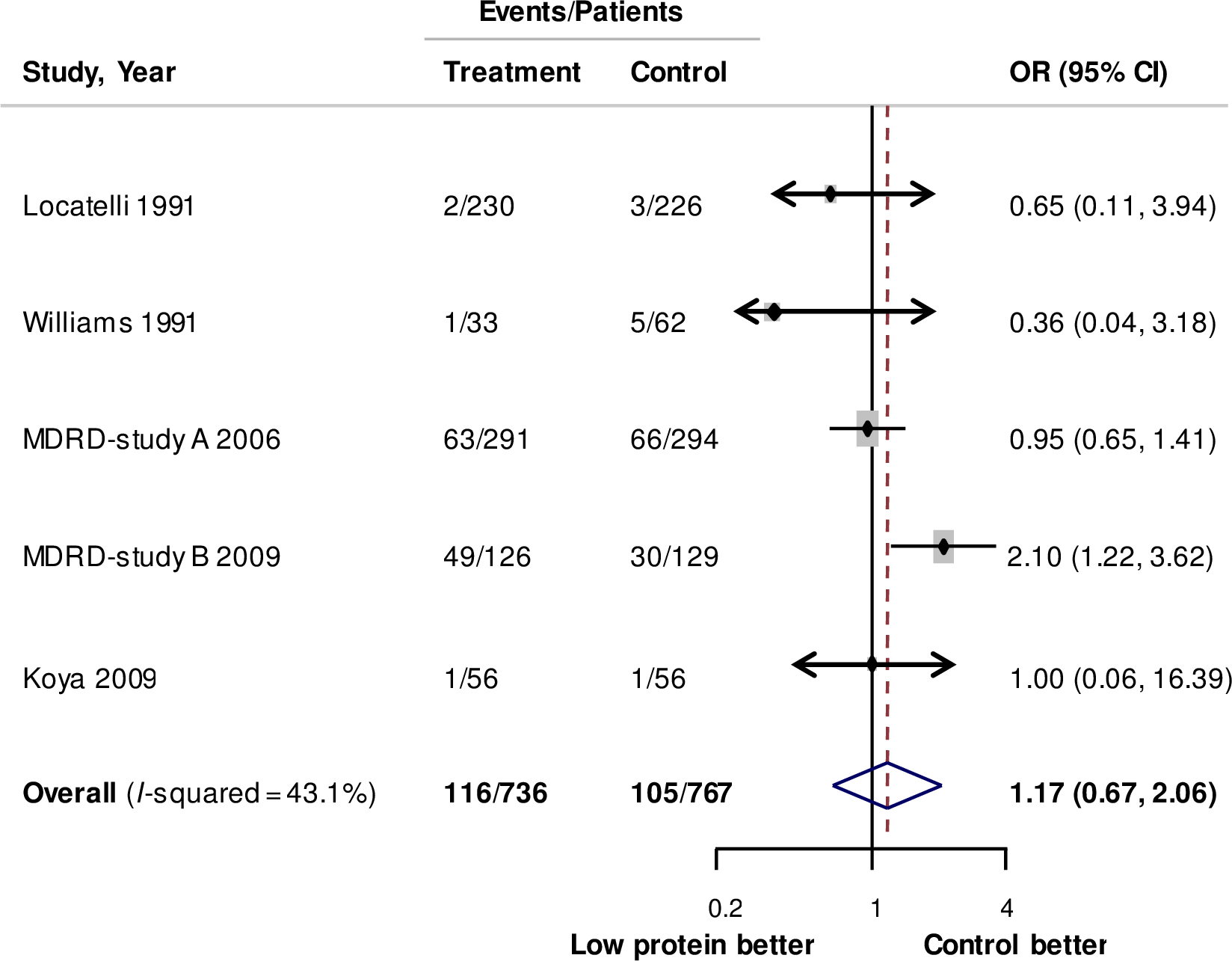

Supplement: S4 Fig — Abbreviations: CI, Confidence interval. (TIF) [file pone.0206134.s007.tif]

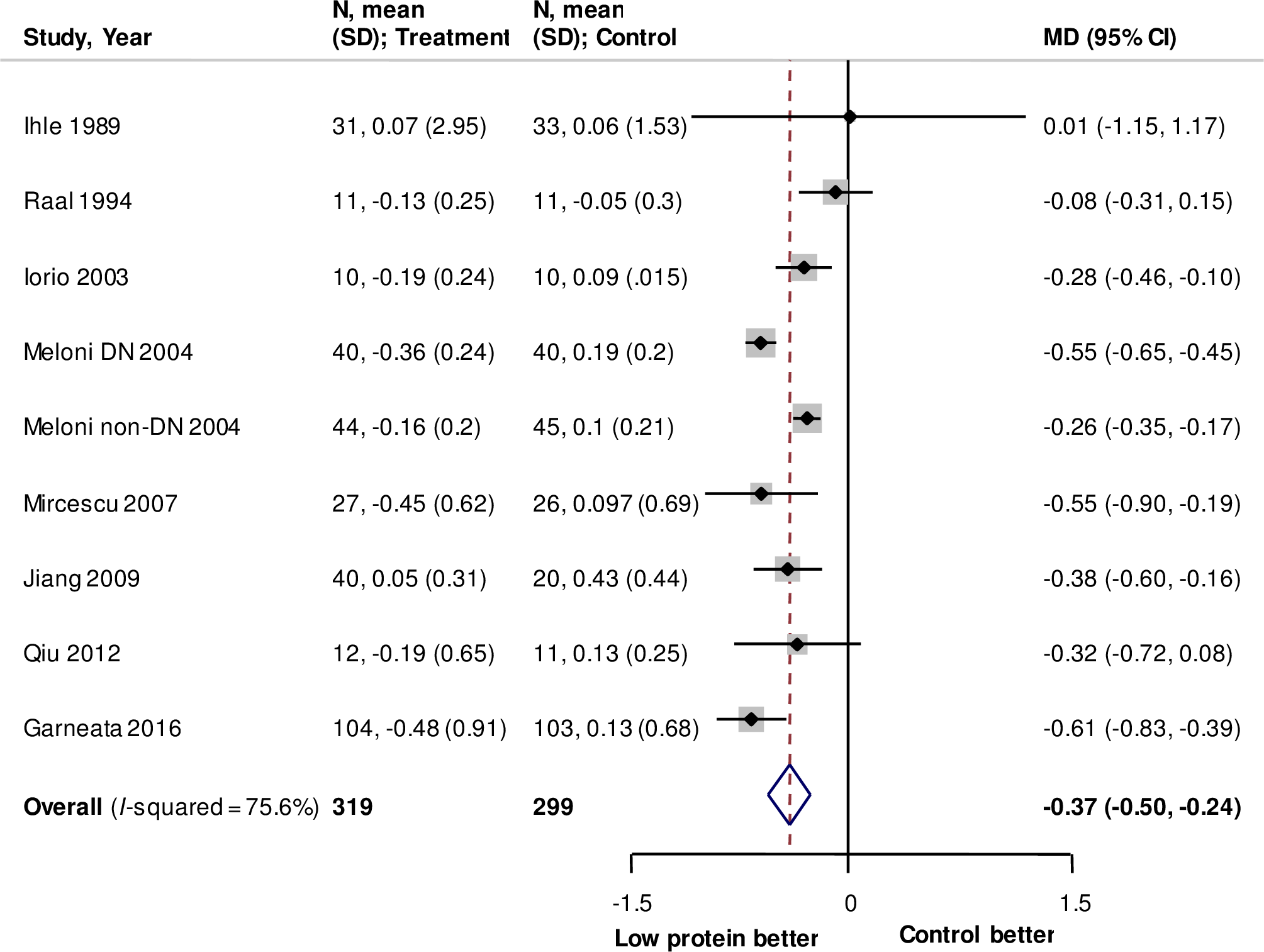

Supplement: S5 Fig — Abbreviations: CI, Confidence interval; MD, mean difference. (TIF) [file pone.0206134.s008.tif]

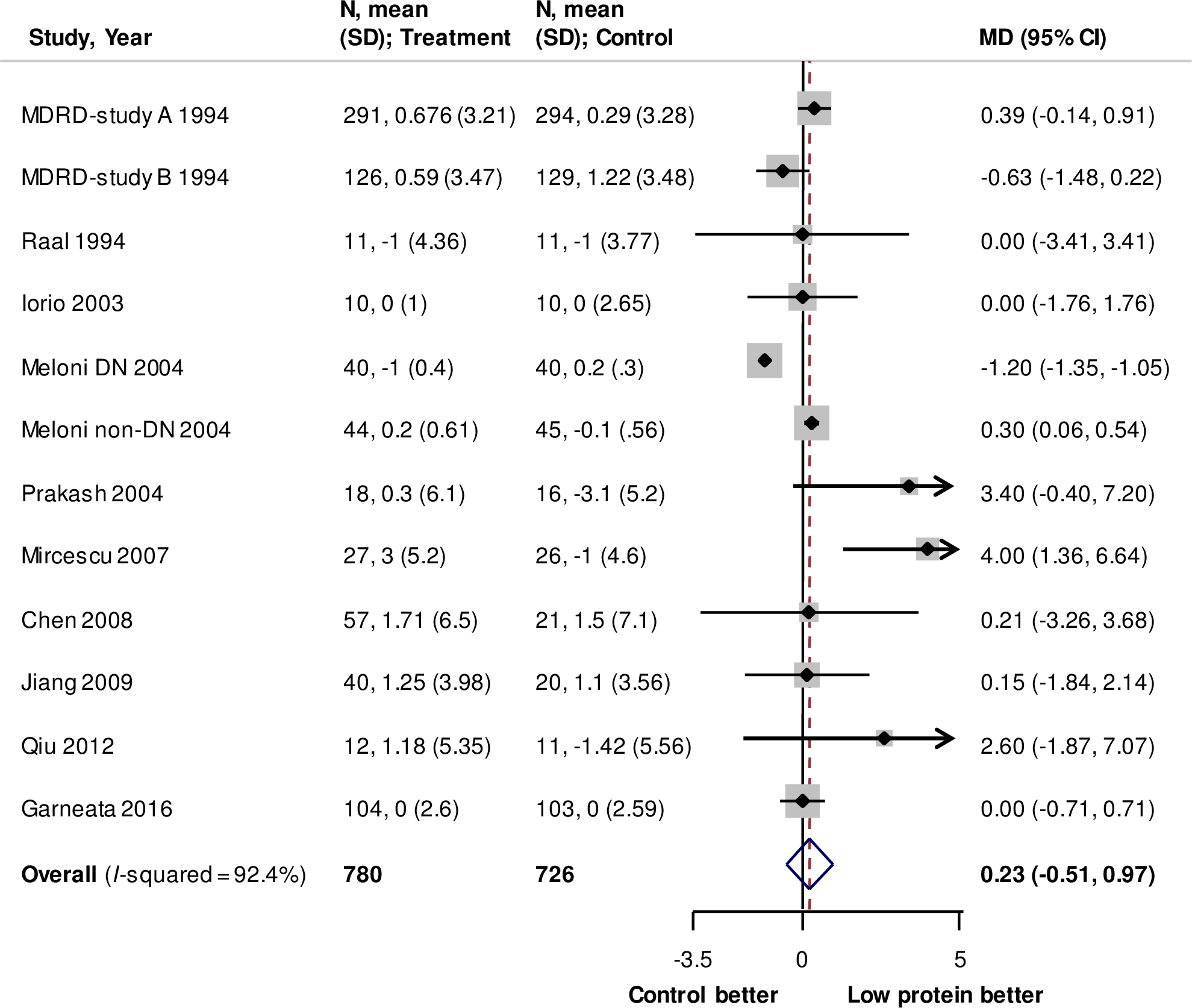

Supplement: S6 Fig — Abbreviations: CI, Confidence interval; MD, mean difference. (TIF) [file pone.0206134.s009.tif]

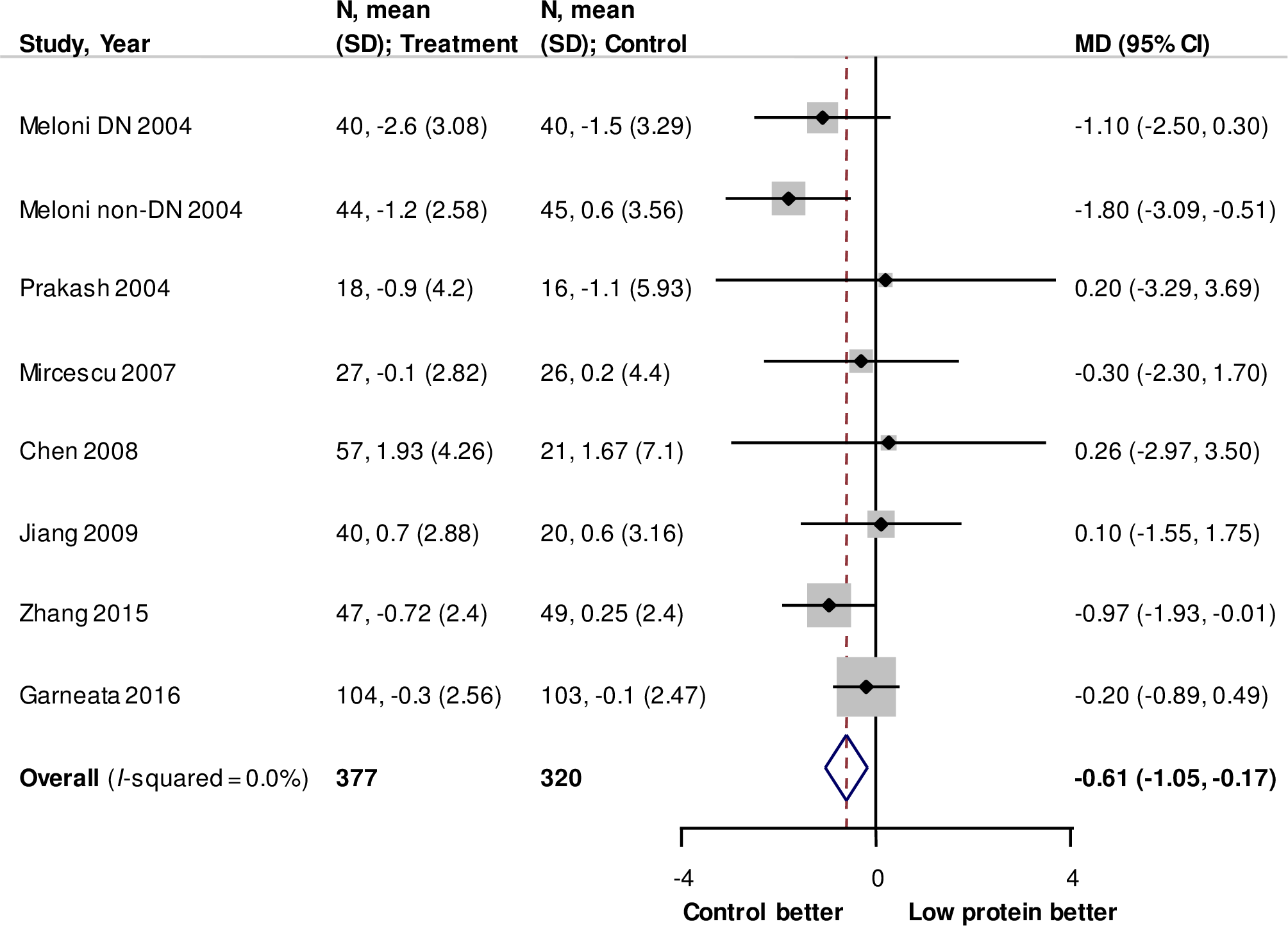

Supplement: S7 Fig — Abbreviations: CI, Confidence interval; MD, mean difference. (TIF) [file pone.0206134.s010.tif]
