# Supplementary material for: Effect of diet protein restriction on progression of chronic kidney disease: A systematic review and meta-analysis
Source: PLoS One. 2018 Nov 7;13(11):e0206134. doi: 10.1371/journal.pone.0206134 (PMC6221301; doi:10.1371/journal.pone.0206134)
Supplement: S1 Table — (DOCX) [file pone.0206134.s011.docx]

**S1 Table: Subgroup Analysis of Kidney Function by Outcome**

| **outcome** | **subgroup** | **No. of trials** | **Sample size** | **Statistic (OR/MD) (95%CI)** | ***P* value for statistic** | ***I*^2^ value** | ***P* value for heterogeneity test** |
| --- | --- | --- | --- | --- | --- | --- | --- |
| **Rate of change in eGFR** | 1. **Mean eGFR (mL/min)** | | | | | | |
|  | < 30 | 7 | 711 | 1.53 (0.44, 2.63) | 0.006 | 73.2% | 0.4 |
|  | 30 - 60 | 6 | 834 | 2.38 (-0.75, 5.51) | 0.1 | 93.3% |  |
|  | > 60 | 1 | 112 | -0.30 (-2.56, 1.96) | 0.8 | - |  |
|  | 1. **Mean proteinuria (g/day)** | | | | | | |
|  | < 1 | 4 | 1100 | 0.61 (0.02, 1.19) | 0.04 | 48.5% | 0.02 |
|  | 1 - 3 | 4 | 303 | 0.60 (-0.85, 2.04) | 0.4 | 0.0% |  |
|  | > 3 | 3 | 136 | 3.19 (-1.64, 8.03) | 0.2 | 97.0% |  |
|  | 1. **Protein intake of experimental group (g/kg/day)** | | | | | | |
|  | < 0.6 | 6 | 633 | 2.43 (0.67, 4.19) | 0.007 | 74.4% | 0.4 |
|  | 0.6 – 0.8 | 5 | 810 | 1.94 (-0.10, 3.99) | 0.2 | 94.9% |  |
|  | > 0.8 | 3 | 214 | 0.07 (-1.78, 1.93) | 0.9 | 0.0% |  |
|  | 1. **DN or not** | | | | | | |
|  | Non-DN | 4 | 413 | 1.91 (0.87, 2.95) | <0.001 | 0.0% | 0.7 |
|  | DN | 5 | 272 | 2.62 (-1.30, 6.55) | 0.2 | 84.7% |  |
|  | 1. **Mean age (years)** | | | | | | |
|  | < 51 | 4 | 199 | 3.03 (-1.06, 7.13) | 0.1 | 95.6% | 0.1 |
|  | ≥51 | 10 | 1458 | 1.07 (0.25, 1.89) | 0.01 | 60% |  |
|  | 1. **Follow-up time (months** ) | | | | | | |
|  | < 12 | 2 | 56 | 8.71 (-3.20, 20.61) | 0.2 | 0.0% | 0.3 |
|  | ≥ 12 | 12 | 1601 | 1.80 (0.72, 2.88) | 0.001 | 88.7% |  |
| **Change of proteinuria** | 1. **Mean eGFR (mL/min)** | | | | | | |
|  | < 30 | 3 | 320 | 0.04 (-0.10, 0.19) | 0.5 | 58.6% | 0.5 |
|  | 30 - 60 | 5 | 342 | -0.96 (-1.68, -0.23) | 0.009 | 81.8% |  |
|  | > 60 | 2 | 208 | -0.34 (-2.58, 1.91) | 0.8 | 95.1% |  |
|  | 1. **Mean proteinuria (g/day)** | | | | | | |
|  | < 1 | 2 | 260 | 0.10 (0.02, 0.18) | 0.02 | 0.0% | 0.09 |
|  | 1 - 3 | 6 | 491 | -0.50 (-1.18, 0.17) | 0.1 | 90.3% |  |
|  | > 3 | 2 | 119 | -1.50 (-2.20, -0.79) | < 0.001 | 0.0% |  |
|  | 1. **Protein intake of experimental group (g/kg/day)** | | | | | | |
|  | < 0.6 | 2 | 260 | 0.10 (0.02, 0.18） | 0.02 | 0.0% | 0.6 |
|  | 0.6 – 0.8 | 5 | 396 | -0.88 (-1.65, -0.12） | 0.02 | 88.7% |  |
|  | > 0.8 | 3 | 214 | -0.38 (-1.75, 0.99） | 0.6 | 89.8% |  |
|  | 1. **DN or not** | | | | | | |
|  | Non-DN | 4 | 477 | -0.32 (-0.77, 0.14) | 0.2 | 95.3% | 0.8 |
|  | DN | 4 | 237 | -0.55 (-1.77, 0.66) | 0.4 | 85.3% |  |
|  | 1. **Mean age (years)** | | | | | | |
|  | < 51 | 2 | 118 | -1.47 (-2.16, -0.78) | < 0.001 | 0.0% | 0.1 |
|  | ≥51 | 8 | 752 | -0.30 (-0.67, 0.07) | 0.1 | 92.5% |  |
|  | 1. **Follow-up time (months )** | | | | | | |
|  | < 12 | 1 | 22 | -1.36 (-3.16, 0.44) | 0.1 | - | 0.5 |
|  | ≥ 12 | 9 | 848 | -0.41 (-0.78, -0.04) | 0.03 | 92.7% |  |
