# Supplementary material for: Effect of diet protein restriction on progression of chronic kidney disease: A systematic review and meta-analysis
Source: PLoS One. 2018 Nov 7;13(11):e0206134. doi: 10.1371/journal.pone.0206134 (PMC6221301; doi:10.1371/journal.pone.0206134)
Supplement: S2 Table — Note: * all the studies included had relatively large sample size more than 50. Kidney failure events was defined as doubling of serum creatinine level or 50% decline in estimated GFR or end-stage renal disease. GFRs expressed in mL/min/1.73m2. Abbreviations: CI, confidence intervals; D-L; DerSimonian-Laird; EB, empirical Bayes; eGFR, estimated glomerular filtration rate; MD, mean difference; n, number of patients; No. number of trials; OR, odds ratio; REML, restricted maximum likelihood;. (DOCX) [file pone.0206134.s012.docx]

**S2 Table: Sensitivity Analyses of Kidney Function**

|  | **Kidney failure events** | | | | |  | | | **eGFR** | | | | | |  | | **proteinuria** | | | | |  |  |
| --- | --- | --- | --- | --- | --- | --- | --- | --- | --- | --- | --- | --- | --- | --- | --- | --- | --- | --- | --- | --- | --- | --- | --- |
|  | **n/No** | **τ^2^** | **OR** | | **95%CI** | |  | | | **n/No** | **τ^2^** | **MD** | | **95%CI** | |  | | **n/No** | **τ^2^** | **MD** | **95%CI** | |  |
| **Base-case** | 1955/9 | 0.2 | 0.59 | | 0.41, 0.85 | |  | | | 1657/14 | 2.4 | 1.85 | | 0.77, 2.93 | |  | | 870/10 | 0.2 | -0.44 | -0.80, -0.08 | |  |
| **Omit sample size < 50** | 1955/9* | 0.2 | 0.59 | | 0.41, 0.85 | |  | | | 1523/9 | 0.1 | 0.49 | | 0.10, 0.87 | |  | | 825/8 | 0.2 | -0.39 | -0.76, -0.02 | |  |
| **Omit follow-up < 12 months** | 1955/9 | 0.2 | 0.59 | | 0.41, 0.85 | |  | | | 1601/12 | 2.4 | 1.80 | | 0.72, 2.88 | |  | | 848/9 | 0.2 | -0.41 | -0.78, -0.04 | |  |
| **Omit Jadad < 3** | 1902/8 | 0.1 | 0.62 | | 0.43, 0.89 | |  | | | 1435/11 | 2.6 | 2.02 | | 0.77, 3.26 | |  | | 648/7 | 0.2 | -0.24 | **-0.69, 0.21** | |  |
| **Different statistical methods** | | | |  | | | | | | | | |  | | | | | | | | | | |
| **D-L** | 1955/9 | 0.2 | 0.59 | | 0.41, 0.85 | |  | 1657/14 | | | 2.4 | 1.85 | | 0.77, 2.93 | |  | | 870/10 | 0.2 | -0.44 | -0.80, -0.08 | |  |
| **REML** | 1955/9 | 0.2 | 0.59 | | 0.36, 0.95 | |  | 1657/14 | | | 4.3 | 2.20 | | 0.61, 3.77 | |  | | 870/10 | 0.6 | -0.49 | **-1.10, 0.12** | |  |
| **EB** | 1955/9 | 0.2 | 0.57 | | 0.35, 0.95 | |  | 1657/14 | | | 4.0 | 2.18 | | 0.63, 3.73 | |  | | 870/10 | 0.6 | -0.49 | **-1.09, 0.11** | |  |

*Note:* * all the studies included had relatively large sample size more than 50. Kidney failure events was deﬁned as doubling of serum creatinine level or 50% decline in estimated GFR or end-stage renal disease. GFRs expressed in mL/min/1.73m^2^.

Abbreviations: eGFR, estimated glomerular filtration rate; n, number of patients; No. number of trials; OR, odds ratio; CI, confidence intervals; MD, mean difference; D-L; DerSimonian-Laird; REML, restricted maximum likelihood; EB, empirical Bayes.
