# Supplementary material for: Effect of diet protein restriction on progression of chronic kidney disease: A systematic review and meta-analysis
Source: PLoS One. 2018 Nov 7;13(11):e0206134. doi: 10.1371/journal.pone.0206134 (PMC6221301; doi:10.1371/journal.pone.0206134)
Supplement: S3 Table — Note: sensitivity analysis A, Results with exclusion of studies with protein intake greater than 0.8 g/kg /day; sensitivity analysis B, Results with exclusion of dialysis patients. (DOCX) [file pone.0206134.s013.docx]

**S3 Table: Post-hoc sensitivity analyses**

| outcome |  | No. of trials | Sample size | Statistic (OR/MD) (95%CI) | *P* value for statistic | *I*^2^ value |
| --- | --- | --- | --- | --- | --- | --- |
| **Kidney failure events** | Base-case | 9 | 1955 | 0.59 (0.41, 0.85) | 0.005 | 56% |
|  | sensitivity analysis A | 8 | 1843 | 0.59 (0.37, 0.82) | 0.004 | 58.8% |
| **Rate of change in eGFR** | Base-case | 14 | 1657 | 1.85 (0.77, 2.93) | 0.001 | 87% |
|  | sensitivity analysis A | 11 | 1443 | 2.13 (0.96, 3.31) | 0.004 | 89% |
| **Change in proteinuria** | Base-case | 10 | 870 | -0.44 (-0.8, -0.08) | 0.02 | 91.9% |
|  | sensitivity analysis A | 7 | 656 | -0.48 (-0.88, -0.09) | 0.02 | 92.9% |
|  | sensitivity analysis B | 9 | 810 | -0.47 (-0.87, -0.07) | 0.02 | 92.6% |
| **All cause death** | Base-case | 5 | 1503 | 1.17 (0.67, 2.06) | 0.6 | 43.1% |
|  | sensitivity analysis A | 4 | 1391 | 1.16 (0.61, 2.19) | 0.7 | 57.2% |
| **Change in phosphorus** | Base-case | 9 | 618 | -0.37 (-0.5, -0.24) | < 0.01 | 75.6% |
|  | sensitivity analysis A | 7 | 516 | -0.37 (-0.48, -0.25) | < 0.01 | 45.7% |
|  | sensitivity analysis B | 8 | 558 | -0.37 (-0.52, -0.22) | < 0.01 | 78.7% |
| **Change in Albumin** | Base-case | 12 | 1506 | 0.23 (-0.51, 0.97) | 0.5 | 92.4% |
|  | sensitivity analysis A | 9 | 1326 | 0.27 (-0.19, 0.73) | 0.3 | 51.5% |
|  | sensitivity analysis B | 10 | 1368 | 0.24 (-0.55, 1.03) | 0.5 | 93.8% |
| **Change in BMI** | Base-case | 8 | 697 | -0.61 (-1.05, -0.17) | 0.007 | 0 |
|  | sensitivity analysis A | 6 | 539 | -0.61 (-1.18, -0.04) | 0.04 | 19% |
|  | sensitivity analysis B | 6 | 559 | -0.74 (-1.27, -0.20) | 0.007 | 14.7% |
